# NanoSimFormer: an end-to-end transformer-based nanopore signal simulator with basecaller guidance

**DOI:** 10.1093/bioinformatics/btag402

**Published:** 2026-06-16

**Authors:** Shaohui Xie, Lulu Ding, Ling Liu, Yew Soon Ong, Jianqiang Li, Zexuan Zhu

**Affiliations:** College of Computer Science and Software Engineering, Shenzhen University, Shenzhen, China; National Engineering Laboratory for Big Data System Computing, Shenzhen University, Shenzhen, China; National Engineering Laboratory for Big Data System Computing, Shenzhen University, Shenzhen, China; School of Artificial Intelligence, Shenzhen University, Shenzhen, China; Guangzhou Institute of Technology, Xidian University, Guangzhou, China; College of Computing and Data Science, Nanyang Technological University, Singapore; National Engineering Laboratory for Big Data System Computing, Shenzhen University, Shenzhen, China; School of Artificial Intelligence, Shenzhen University, Shenzhen, China; National Engineering Laboratory for Big Data System Computing, Shenzhen University, Shenzhen, China; School of Artificial Intelligence, Shenzhen University, Shenzhen, China

## Abstract

**Motivation:**

High-fidelity simulation of nanopore sequencing signals is critical for rigorous benchmarking and validation of the nanopore signal processing pipeline. However, existing signal simulators often fail to capture the non-linear dynamics of nanopore current signals, relying on static pore models or lacking optimization objectives tied to basecalling, resulting in synthetic signals with low basecalling accuracy and fidelity.

**Results:**

We introduce NanoSimFormer, an end-to-end Transformer-based signal simulator that integrates basecaller guidance during training to generate high-fidelity nanopore signals. NanoSimFormer achieves a median basecalling accuracy exceeding 99% and Q-scores above 22.8 for Oxford Nanopore Technologies’ latest DNA R10.4.1 and direct RNA sequencing, closely mirroring real experimental baselines. It faithfully recapitulates experimental variant calling performance across the five human samples, achieving F1-scores of 0.9953–0.9973 and 0.7862–0.8612 for single-nucleotide polymorphisms and small indels detections, respectively. Compared with previous simulators, NanoSimFormer also substantially reduces false positives in homopolymer and short tandem repeat regions. NanoSimFormer-derived reads enable high-quality de novo bacterial assembly with consensus error rates below one mismatch per 100 kbp and maintain high correlations with experimental abundance in metagenomic and transcriptomic datasets.

**Availability and implementation:**

NanoSimFormer is freely available on GitHub at: https://github.com/BioinfoSZU/NanoSimFormer.

## 1 Introduction

Nanopore sequencing has revolutionized genomic and transcriptomic research by enabling direct and real-time analysis of long nucleic acid molecules without amplification (Deamer *et al*. 2016). It has been widely used for the detection of single-nucleotide polymorphisms (SNPs) and complex structural variants (SVs) (De Coster *et al*. 2019, Shafin *et al*. 2021), de novo genome assembly (Jain *et al*. 2018), epigenetic modification calling (Simpson *et al*. 2017), and transcript isoform profiling (Chen *et al*. 2025). Nanopore sequencing also offers a new opportunity to clinical diagnostics where real-time decision-making is required (Loose *et al*. 2016, Massaiu *et al*. 2025). Nevertheless, nanopore sequencing has long been constrained by its relatively high error rate. Recently, the field has witnessed a substantial technological advancement with the introduction of Oxford Nanopore Technologies’ (ONT) R10.4.1 flow cell, which features a dual-reader-head pore architecture, along with the v14 sequencing kit that incorporates the high-processivity E8.2.1 helicase enzyme (Dorey and Howorka 2024). Coupled with the latest official basecaller Dorado (https://github.com/nanoporetech/dorado) and the released Transformer-based super-accuracy basecalling models (v5), this system achieves a new standard of performance, delivering DNA basecalling accuracy exceeding 99% (Q20+) (https://nanoporetech.com/platform/accuracy). The ONT direct RNA sequencing (DRS) ecosystem has also advanced with the RNA004 chemistry, leveraging novel motor proteins and pore architectures to deliver significantly enhanced accuracy and throughput for native RNA characterization (Hewel *et al*. 2025).

With the continued evolution of nanopore sequencing technologies, the need for high-fidelity simulation of sequencing signal data has grown increasingly important. In silico data generation enables the creation of scalable datasets under controlled conditions, facilitating rigorous benchmarking of analysis pipelines. It also supports the debugging, optimization, and validation of new algorithms without the confounding effects of experimental variability (Escalona *et al*. 2016). Historically, long-read simulators such as NanoSim (Yang *et al*. 2017), Trans-NanoSim (Hafezqorani *et al*. 2020), and Badread (Wick 2019) have played a pivotal role in modeling sequencing error profiles for downstream analyses. However, these tools generate only nucleotide sequences and do not simulate the raw ionic current signals that are fundamental to nanopore sequencing. Modeling these signals has been shown to substantially enhance simulation fidelity (Li *et al*. 2018). DeepSimulator (Li *et al*. 2018, 2020) pioneered signal-level simulation by employing a context-dependent model based on a bi-directional long short-term memory architecture. Subsequently, NanosigSim (Chen *et al*. 2020) incorporated bidirectional gated recurrent units to better characterize signal noise. More recently, Squigulator (Gamaarachchi *et al*. 2024) introduced a computationally efficient framework leveraging static k-mer pore models combined with empirical statistical distributions, while seq2squiggle (Beslic *et al*. 2024) adopted a non-autoregressive feed-forward Transformer to learn signal generation directly from training data.

Despite these advancements, the existing nanopore signal simulation methods suffer from several critical limitations. First, they were developed independently of the basecalling process. Without feedback from the basecaller, simulated signals may resemble real data visually yet fail to preserve features essential for accurate basecalling, effectively decoupling signal generation from its ultimate objective. Second, most simulators, including DeepSimulator, NanosigSim, and Squigulator, rely on predefined k-mer pore models to derive event-level signals. This dependence on static, discretized representations constrains their ability to capture the complex, nonlinear dynamics of nanopore currents and to model long-range dependencies between signals and nucleotide sequences. Moreover, the development of data simulators cannot keep up with the pace of sequencing technologies. Although Squigulator and seq2squiggle support the latest ONT DNA R10 sequencing, their modeling of the latest flow cells remains coarse, resulting in median read accuracies (e.g. 91.91–95.29% for seq2squiggle) that fall substantially short of real experimental performance. In addition, there is a critical lack of efficient simulation tools capable of modeling the latest RNA004 chemistry for DRS.

To address these limitations, here we propose NanoSimFormer, an end-to-end Transformer-based nanopore signal simulator that incorporates basecaller-guided training to generate high-fidelity signals tailored to the latest ONT DNA R10.4.1 sequencing and DRS. NanoSimFormer adopts a non-autoregressive architecture comprising a Transformer-based sequence encoder and lightweight feedforward neural networks that predict per-base signal mean and noise standard deviation to model complex contextual dependencies and nonlinear signal dynamics. Unlike prior approaches that rely solely on event-level signal reconstruction losses, NanoSimFormer employs a frozen basecalling model as a discriminator and is trained using a multi-objective optimization strategy to generate signals explicitly tuned for accurate basecalling. This basecaller-guided training strategy is the central innovation that distinguishes NanoSimFormer from the prior Transformer-based simulator seq2squiggle (Beslic *et al*. 2024), which shares a similar encoder architecture and signal generation approach but lacks basecalling feedback. We systematically evaluated NanoSimFormer against state-of-the-art simulators, seq2squiggle and Squigulator, across diverse human, bacterial, and fungal datasets. NanoSimFormer demonstrated superior signal fidelity, achieving median read accuracies of 98.74–99.65% and Q-scores (>22.8), closely matching real experimental baselines for both ONT DNA R10.4.1 and DRS. It accurately recapitulated experimental variant-calling performance, attaining F1-scores of 0.9953–0.9973 for SNPs detection and 0.7862–0.8612 for small indels detection across the five human R10.4.1 datasets, while markedly reducing false positives in homopolymer and short tandem repeat regions. NanoSimFormer-derived reads supported high-quality de novo bacterial assembly with consensus error rates below one mismatch per 100 kbp, comparable to the real experimental assemblies. The model was also shown to preserve fungal metagenomic community composition and human transcriptomic profiles, demonstrating strong correlation with experimental abundance estimates. With tunable parameters controlling variance of the additional read-level amplitude noise and event-duration, NanoSimFormer provides a flexible framework for generating simulated datasets of customizable quality across diverse benchmarking scenarios. The ablation study by removing basecaller guidance from NanoSimFormer degrades its performance to a level comparable to seq2squiggle, confirming that basecaller-guided training is the primary driver of signal fidelity.

## 2 Materials and methods

### 2.1 Overview

**Figure 1:**
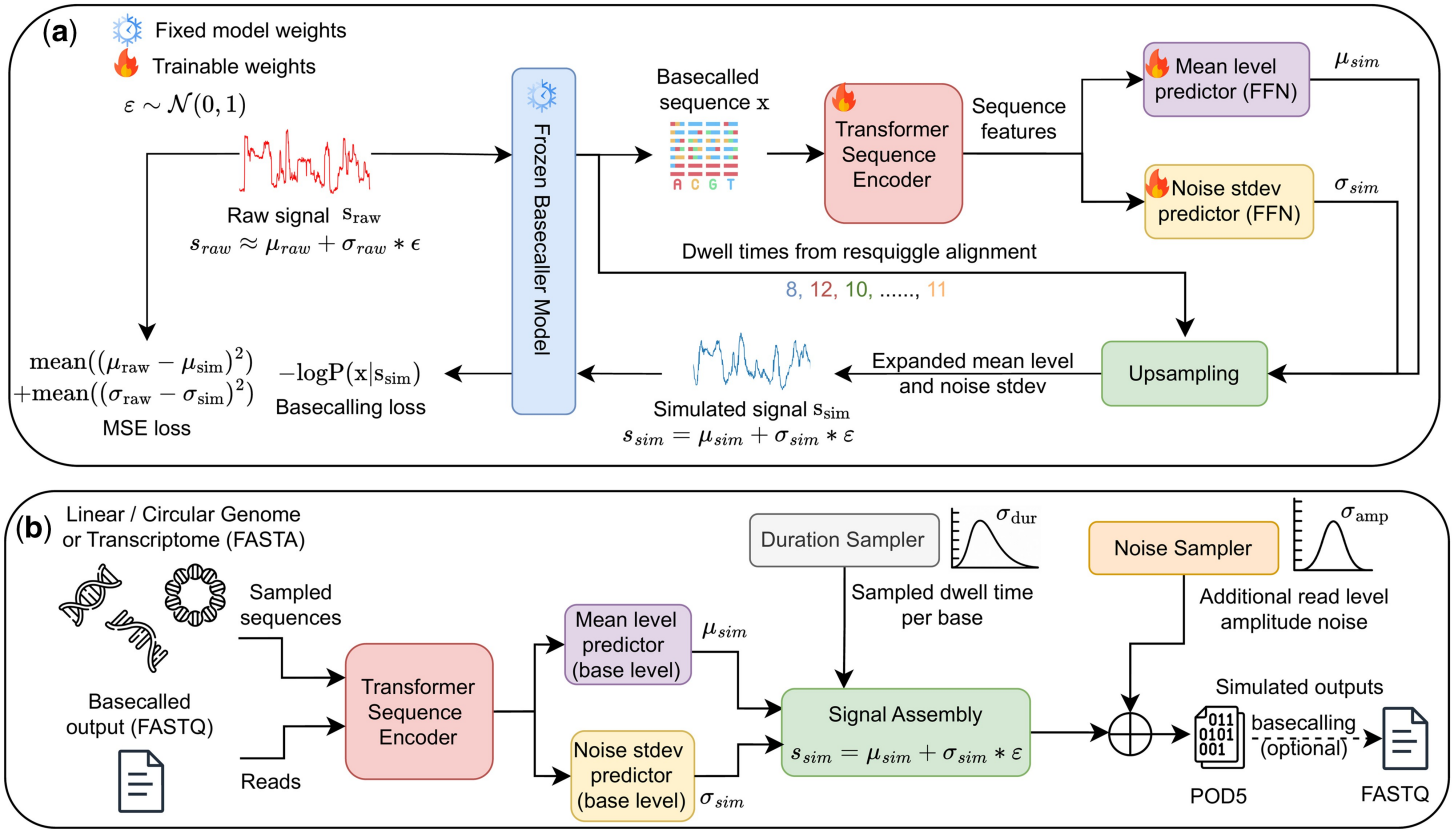
Overview of the NanoSimFormer framework. (a) Training workflow incorporating basecaller guidance. A frozen basecaller is utilized (blue box with snowflake icon) to derive the basecalled sequence (*x*), followed by a resquiggle alignment to estimate per-base dwell times (illustrated with different colors to indicate the signal duration associated with each base) from the raw signal ($s_{\text{raw}}$). The trainable components (flame icon) comprise the Transformer sequence encoder and two lightweight FFNs for per-base mean signal level ($\mu_{\text{sim}}$) and noise standard deviation ($\sigma_{\text{sim}}$) prediction. The simulated signal is constructed using a parametric Gaussian approach: $s_{\text{sim}}=\mu_{\text{sim}}+\sigma_{\text{sim}}\cdot \varepsilon$ and optimized using both event-level MSE losses and a basecalling loss. (b) Simulation workflow. Target sequences are sampled from a reference (FASTA) or directly from basecalled reads (FASTQ). The trained Transformer encoder extracts sequence features, and two FFN heads predict per-base mean signal and noise standard deviation. A duration sampler generates event durations (with standard deviation $\sigma_{\text{dur}}$), replacing the resquiggle-derived dwell times used in training. The per-base predictions are expanded according to sampled dwell times and assembled into the signal. A noise sampler adds additional per-read Gaussian noise (standard deviation $\sigma_{\text{amp}}$) to produce the final simulated signals in POD5 format. These signals can subsequently be basecalled (optionally) to produce sequence reads in FASTQ format. Icons used in this figure were sourced from Flaticon (https://www.flaticon.com).

NanoSimFormer utilizes an end-to-end Transformer-based architecture that integrates basecaller guidance (Fig. 1). During training (Fig. 1a), a frozen basecalling model processes experimental raw signals to derive the nucleotide sequences. To ensure the consistency between training and inference, sequences used in training are filtered using stringent criteria, including a minimum accuracy of 99% and a minimum quality score of Q20. A reference-anchored signal-to-sequence alignment refinement (“resquiggle”) is then performed to obtain precise per-base dwell times (the number of raw signal samples associated with each nucleotide). The nucleotide sequences are mapped into high-level context features by a Transformer sequence encoder. Two lightweight feedforward neural networks (FFNs) then predict the per-base mean signal level $\mu_{\text{sim}}$ and per-base noise standard deviation $\sigma_{\text{sim}}$ from these features. Each base’s signal segment is constructed parametrically by expanding $\mu_{\text{sim}}$ and $\sigma_{\text{sim}}$ according to the dwell time and sampling Gaussian noise: $s_{\text{sim}}=\mu_{\text{sim}}+\sigma_{\text{sim}}\cdot \varepsilon$, where ε∼N(0,1). The model is trained using a multi-objective loss combining a mean squared error (MSE) term for base-level mean signal and noise standard deviation prediction and a basecalling loss through a frozen basecalling model to ensure fidelity at both the signal and sequence levels. During simulation (Fig. 1b), the dwell times are randomly sampled from a duration sampler, and an optional per-read amplitude noise is applied to the assembled signal. NanoSimFormer supports diverse input formats, including linear/circular genome or transcriptome references (FASTA) and experimental basecalled reads (FASTQ).

### 2.2 Model architecture and training

NanoSimFormer employs an end-to-end Transformer-based non-autoregressive architecture to generate high-fidelity signals $s_{\text{sim}}\in R^{T}$ from an input nucleotide sequence $x\in \Sigma^{L}$, where *L* is the sequence length, *T* is the number of signal timepoints, and Σ={A,C,G,T} is the alphabet of nucleotide bases.

The *sequence encoder* of NanoSimFormer is designed to extract rich contextual representations from the nucleotide sequence (Fig. S1a, available as supplementary data at *Bioinformatics* online). The input sequence *x* is first embedded into a high-dimensional space via an embedding layer. Specifically, each symbol is mapped to a vector using a learnable lookup table, resulting in a sequence of embeddings $h\in R^{L\times d}$, where *d* is the feature dimension. These embeddings are then processed by *N* stacked Transformer encoder blocks (N=12 in our implementation). Each block comprises a Multi-Head Self-Attention (MHSA) module and a gated FFN, both with layer normalization and residual connections. Given an input feature sequence h, the MHSA mechanism projects inputs into queries $q_{h}^{\left(e\right)}$, keys $k_{h}^{\left(e\right)}$, and values $v_{h}^{\left(e\right)}$ for each attention head e∈{1,…,E}, where *E* is the number of heads. Specifically:


(1) $$\begin{array}{c} q_{h}^{\left(e\right)}=hQ_{h}^{\left(e\right)},k_{h}^{\left(e\right)}=hK_{h}^{\left(e\right)},\text{ and }v_{h}^{\left(e\right)}=hV_{h}^{\left(e\right)} \end{array}$$

Where $Q_{h}^{\left(e\right)},K_{h}^{\left(e\right)},V_{h}^{\left(e\right)}\in R^{d\times d_{e}}$ denote the learnable projection matrices that transform the signal feature sequence into queries, keys, and values, respectively. $d_{e}$ is the head dimension ($d_{e}$ = 64). Additionally, rotary positional embeddings (Su *et al*. 2024) are then applied to the queries $q_{h}^{\left(e\right)}$ and keys $k_{h}^{\left(e\right)}$ to incorporate relative positional information. Afterward, the self-attention weight $\alpha_{h}^{\left(e\right)}$ and the output representations $o_{h}^{\left(e\right)}$ are computed for each head as follows:


(2) $$\begin{array}{c} \alpha_{h}^{\left(e\right)}=\text{Softmax}\left(\frac{q_{h}^{\left(e\right)}{\left(k_{h}^{\left(e\right)}\right)}^{⊤}}{\sqrt{d_{e}}}\right)\\ o_{h}^{\left(e\right)}=\alpha_{h}^{\left(e\right)}v_{h}^{\left(e\right)} \end{array}$$

The outputs from all attention heads are concatenated and projected to the original dimension:


(3) $$\begin{array}{c} \text{MHSA}(h)=\text{Concat}(o_{h}^{\left(1\right)},\ldots ,o_{h}^{\left(E\right)})W_{O} \end{array}$$

Where $W_{O}\in R^{(E\cdot d_{e})\times d}$ is a learnable output projection matrix. The FFN network after the MHSA module employs a Swish Gated Linear Unit (SwiGLU) variant (Shazeer 2020). The SwiGLU introduces nonlinearity, dimension expansion, and feature selection into the encoder layer. Specifically:


(4) $$\begin{array}{c} \text{SwiGLU}(h)=(\text{Swish}(hW_{g})⊙(hW_{l}))W_{f} \end{array}$$

Where $W_{g},W_{l}\in R^{d\times 4d}$ denote the learnable projection matrices that expand the dimension, whereas $W_{f}\in R^{4d\times d}$ shrinks the dimension back to *d*.

Sequence features from the encoder are passed to two lightweight FFN heads, analogous to the prediction modules used in seq2squiggle (Fig. S1b, available as supplementary data at *Bioinformatics* online). The *mean level predictor* maps each base’s *d*-dimensional feature vector to a scalar mean signal level $\mu_{\text{sim}}$ through two dense layers with Swish activation. The *noise standard deviation predictor* uses the same architecture, with an additional Softplus activation to ensure a positive output $\sigma_{\text{sim}}$. During training, event durations are derived from the dwell times obtained from the resquiggle alignment using the ont-remora (https://github.com/nanoporetech/remora) API. For each base $x_{t}$ with dwell time $e_{t}$, the predicted $\mu_{\text{sim}}^{\left(t\right)}$ and $\sigma_{\text{sim}}^{\left(t\right)}$ are each replicated $e_{t}$ times. The simulated signal is then constructed parametrically as:


(5) $$s_{\text{sim}}^{\left(i\right)}=\mu_{\text{sim}}^{\left(t\right)}+\sigma_{\text{sim}}^{\left(t\right)}\cdot \varepsilon^{\left(i\right)},\;\varepsilon^{\left(i\right)}∼N(0,1)$$

for each signal sample *i* within the dwell interval of base $x_{t}$. During simulation, event durations are generated by a duration sampler. For both DNA R10.4.1 and RNA004 simulations, event durations follow a Gamma distribution with a fixed mean (12 for R10.4.1, corresponding to the average dwell time at 400 bps and 5 kHz sampling rate; 30 for RNA004, corresponding to the average dwell time at 130 bps and 4 kHz sampling rate) and a tunable standard deviation $\sigma_{\text{dur}}$. This design effectively captures the stochastic variability of enzymatic motor stepping speed and better models the long-tailed dwell-time distribution observed in nanopore sequencing. After signal construction, an optional zero-mean per-read Gaussian amplitude noise with standard deviation $\sigma_{\text{amp}}$ is added to the simulated signal at the picoampere scale. This read-level noise is distinct from the per-base noise $\sigma_{\text{sim}}$ learned during training, and serves as a separate, user-configurable parameter to model the stochastic variation in experimental conditions across reads. A more detailed analysis of the impact of these two tunable parameters is provided in Section 3.4.

The model parameters θ are estimated by minimizing a multi-objective loss function $L_{\mathrm{total}}$ that enforces realism in both signal and sequence levels:


(6) $$\begin{array}{c} L_{\text{total}}=L_{\text{MSE}}^{\mu }+L_{\text{MSE}}^{\sigma }+L_{\text{Basecalling}}\\ L_{\text{MSE}}^{\mu }=\frac{1}{L}\sum_{t=1}^{L}{\left(\mu_{\text{sim}}^{\left(t\right)}-\mu_{\text{raw}}^{\left(t\right)}\right)}^{2}\\ L_{\text{MSE}}^{\sigma }=\frac{1}{L}\sum_{t=1}^{L}{\left(\sigma_{\text{sim}}^{\left(t\right)}-\sigma_{\text{raw}}^{\left(t\right)}\right)}^{2}\\ L_{\text{Basecalling}}=-\text{log}\;P(x|s_{\text{sim}};\phi ) \end{array}$$

The MSE loss $L_{\text{MSE}}^{\mu }$ and $L_{\text{MSE}}^{\sigma }$ quantify the event-level discrepancy between the predicted and target per-base mean signal level and noise standard deviation, respectively. The training targets $\mu_{\text{raw}}$ and $\sigma_{\text{raw}}$ are derived from the resquiggle alignment and correspond to the empirical mean signal level and standard deviation of the raw experimental signal within the dwell interval of each base. The basecalling loss $L_{\text{Basecalling}}$ is defined as the negative log-likelihood of the nucleotide sequence *x* given the simulated signal. This is implemented using connectionist temporal classification (CTC) loss (Graves *et al*. 2006) via a pre-trained and frozen basecaller (the Dorado v5 super-accuracy model, parameterized by ϕ):


(7) $$-\text{log}\;P(x|s_{\text{sim}};\phi )=-\text{log}\;\sum_{\pi \in B^{-1}(x)}\prod_{t=1}^{T^{\prime }}P(\pi_{t}|s_{\text{sim}};\phi )$$

Where $T^{\prime }$ denotes the number of output frames after the basecaller’s encoder, and $B^{-1}(x)$ is the set of all valid CTC paths that collapse to *x* under the many-to-one map function B, which removes repeated labels and blanks. The sum over all valid paths is computed efficiently using the CTC forward–backward algorithm implemented in the official ont-koi package, which provides a CUDA-based implementation for GPU-accelerated training. All computations are performed at the batch level with a fixed batch size. At each training step, a batch of simulated signal chunks is passed through the frozen basecaller in a single forward pass, and the CTC loss is computed over the entire batch simultaneously. This batched design maximizes GPU utilization and improves computational efficiency. Although the basecaller remains fully differentiable, its parameters ϕ are frozen by setting requires_grad=False, which prevents PyTorch from computing or storing gradients for the basecaller parameters. During backpropagation, the gradient of the CTC loss with respect to the simulated signal is propagated through the frozen basecaller to update the trainable NanoSimFormer modules, while the basecaller parameters act as fixed constants in the chain rule. Freezing the basecaller also substantially reduces memory consumption, as no gradient buffers or optimizer states are allocated for its parameters, lowering per-parameter memory usage by approximately fourfold compared with full basecaller training. In addition, mixed-precision training is used to further improve throughput. Overall, this basecaller-guided objective directly penalizes simulated signals that cannot be accurately decoded and provides a stronger structural regularization during training, which is the key innovation distinguishing NanoSimFormer from prior simulators. More details of the training configuration are listed in Table S1, available as supplementary data at *Bioinformatics* online.

### 2.3 Experimental setup

#### 2.3.1 Datasets

The NanoSimFormer DNA model was trained on reads from chromosome 1 of a publicly available HG002 R10.4.1 sample (ENA accession: ERR12997168) (Wong *et al*. 2024). The sequencing reads were first basecalled using the Dorado super-accuracy model (v5.0.0) with the options—emit-moves to enable “moves” output, then aligned to the perfectly accurate diploid reference genome from the T2T-HG002 Q100 project (Hansen *et al*. 2025) to filter low-quality reads (minimum accuracy 99%, minimum quality Q20). To obtain precise per-base signal-to-sequence correspondence, a reference-anchored signal-to-sequence alignment (“resquiggle”) was performed using the ont-remora API, which takes the aligned BAM file (containing MD and move-table tags) together with the raw signal in POD5 format to produce a per-base signal index mapping. Each training chunk consists of a contiguous signal segment of fixed length, paired with the corresponding variable-length nucleotide subsequence determined by the resquiggle mapping. The nucleotide sequence length within each chunk is zero-padded to the maximum allowed sequence length (e.g. 500 bp) when shorter, and a self-attention mask is used during training to exclude the padded positions. Subsequently, thirteen million high-accuracy chunks with 5,000 signal samples were randomly selected from the dataset for training. The NanoSimFormer RNA model was trained on reads from Universal Human Reference (UHR) RNA004 sample (ENA accession: ERR12997170) (Wong *et al*. 2024). The sequencing reads were first basecalled using the Dorado super-accuracy RNA model (v5.0.0), then aligned to the GENCODE (v46) human reference transcriptome. The dataset preparation for RNA004 was similar in DNA dataset. Finally, five million chunks with 12,000 signal samples were randomly selected for RNA model training.

For simulation evaluation, we employed diverse samples and species strictly independent of the training set, including:

- *Homo sapiens* HG002 chromosome 22 reads (Wong *et al*. 2024), with simulation performed using the diploid chromosome 22 reference.
- Five bacterial species (*E. coli*, *K. pneumoniae*, *M. morganii*, *P. aeruginosa*, and *P. mirabilis*) with ground-truth genomes assembled by Hybracter using both ONT and Illumina data (Prior *et al*. 2025).
- The ATCC Mycobiome Genomic DNA Mix (MSA-1010) metagenomic dataset comprising ten fungal strains (https://registry.opendata.aws/ont-open-data), simulated using basecalled reads (FASTQ) as input to preserve the natural concentration and relative abundance of the fungal community.
- *Homo sapiens* HG002 RNA004 chromosome 22 reads (Zheng *et al*. 2025), with the transcriptome reference derived from the HG002 diploid assembly annotation.
- Four additional independent human DNA R10.4.1 datasets (HG001, HG003, HG004, and HG005) from the ONT Open Data project, with corresponding diploid references derived from the GIAB truth set, were used to evaluate cross-dataset generalization in signal-level fidelity and small variants detection.

More details of the datasets can be found in Supplementary Tables S2 and S3, available as supplementary data at *Bioinformatics* online.

#### 2.3.2 Benchmark pipelines

NanoSimFormer was benchmarked against seq2squiggle (v0.3.4) (Beslic *et al*. 2024) and Squigulator (v0.4.0) (Gamaarachchi *et al*. 2024), the leading simulators supporting R10.4.1 chemistry. For DRS simulation, only Squigulator was compared as it is the sole alternative supporting RNA004. Squigulator and seq2squiggle were run using their default configuration. All simulators run with a mean read length configuration estimated from real experimental data to ensure a similar sequencing depth as the experimental data. For reference-based simulation, NanoSimFormer selected an exponential distribution or a statistical model derived from the human HG002 sample as the read length distribution, based on an exploration of real experimental data. More details of the simulation settings can be found in Table S4, available as supplementary data at *Bioinformatics* online. Real experimental sequencing data were included in all comparisons to serve as the ground-truth baseline. All simulated and real experimental signals were basecalled using Dorado with the super-accuracy models (v5.0.0) and aligned to the respective reference using minimap2 (v2.28-r1209) (Li 2018) with the options “-axlr: hq—secondary=no—eqx” to disable secondary alignments. The unaligned reads and the supplementary alignments were further excluded from the evaluation. The read accuracy is used as the primary metric for evaluating the basecalling performance:


(8) $$\text{accuracy}=\frac{N_{\text{match}}}{N_{\text{match}}+N_{\text{mis}}+N_{\text{ins}}+N_{\text{del}}}$$

Where $N_{\text{match}}$, $N_{\text{mis}}$, $N_{\text{ins}}$, and $N_{\text{del}}$, extracted from the CIGAR string, represent the numbers of matching bases, mismatches, insertions, and deletions of a read, respectively. The error (mismatch, insertion, and deletion) rate per read can be defined as:


(9) $${\text{err}}_{\text{mis}/\text{ins}/\text{del}}=\frac{N_{\text{mis}/\text{ins}/\text{del}}}{N_{\text{match}}+N_{\text{mis}}+N_{\text{ins}}+N_{\text{del}}}$$

To quantify signal-level fidelity independent of basecalling, we computed the Dynamic Time Warping (DTW) distance (Giorgino 2009) between simulated and experimental raw signals over entire reads on pico-ampere scale, using the symmetric2 step pattern with Sakoe–Chiba banded alignment constraint (window fraction = 0.25, i.e., the allowed warping band width is 25% of the longer signal length). DTW distances were normalized by the maximum of the two compared signal lengths to ensure comparability across reads of different lengths. The DTW evaluation was performed across five human DNA R10.4.1 datasets (HG001–HG005) to assess cross-dataset generalization. For each dataset, 20,000 reads with sequence length ≤6000 bp and signal lengths <200,000 were randomly sampled to balance computational efficiency with statistical robustness.

To rigorously assess the utility of simulated signals for downstream analysis, we established comprehensive pipelines for several representative tasks. For variant calling, we followed procedures established in (Beslic *et al*. 2024, Gamaarachchi *et al*. 2024). For DNA R10.4.1 small variants calling, we evaluated all simulators on five human genome samples (HG001–HG005). Both real experimental and simulated reads derived from the corresponding diploid chromosome 22 reference genome were used in this evaluation. For RNA004 variant calling, both real experimental and simulated reads derived from the HG002 transcriptome were used. Basecalled reads were aligned to the GRCh38 reference genome using minimap2 (v2.28-r1209) (Li 2018). Small variants (SNPs and small indels ≤30 bp) were identified using Clair3 (v1.2.0) (Zheng *et al*. 2022) for DNA R10.4.1 reads and Clair3-RNA (v0.2.2) (Zheng *et al*. 2025) for DRS RNA004 reads. The variant calling results were filtered to exclude low-quality calls (QUAL > 0 and DP > 1) using bcftools and subsequently benchmarked against the Genome in a Bottle (GIAB) (Zook *et al*. 2016) v3.3.2 high-confidence variant set using RTG tools (v3.13) (Cleary *et al*. 2015). Structural variants (SVs) were detected using Sniffles2 (v2.7.0) (Smolka *et al*. 2024) with tandem repeat annotations enabled. The SV call sets were evaluated against the GIAB HG002 Structural Variants Draft Benchmark Set (V0.019–20241113) using Truvari (v5.4.0) (English *et al*. 2022). To assess the utility of the simulated reads for genomic assembly, de novo assemblies were performed on the five bacterial datasets using nanoMDBG (v1.2) (Benoit *et al*. 2026), polished with Medaka (v2.1.1) (https://github.com/nanoporetech/medaka) and evaluated against ground-truth references using QUAST (v5.2.0) (Gurevich *et al*. 2013) to quantify metrics including genome fraction, indels, and mismatch rates. For metagenomic community structure preservation, the relative abundance of the ten constituent fungal strains in the MSA-1010 mock community was estimated by aligning basecalled reads to the MSA-1010 reference genome with minimap2, and the correlation between simulated and real experimental abundance profiles was quantified using the Pearson correlation coefficient. For transcriptomics simulation, isoform identification and quantification were performed using ESPRESSO (v1.6.0) (Gao *et al*. 2023) on the HG002-RNA004 dataset, which categorizes reads into full splice match (FSM), incomplete splice match (ISM), novel in catalog (NIC), novel not in catalog (NNC), single-exon (SEX), and not completely determined (NCD) according to the alignment status around known canonical splice junctions. Transcript-level expression correlation between simulated and real experimental data was quantified using the Pearson correlation coefficient. Detailed tool commands and configurations in downstream analysis are provided in Supplementary Note S1.

## 3 Results

### 3.1 Basecalling accuracy and signal fidelity

**Figure 2:**
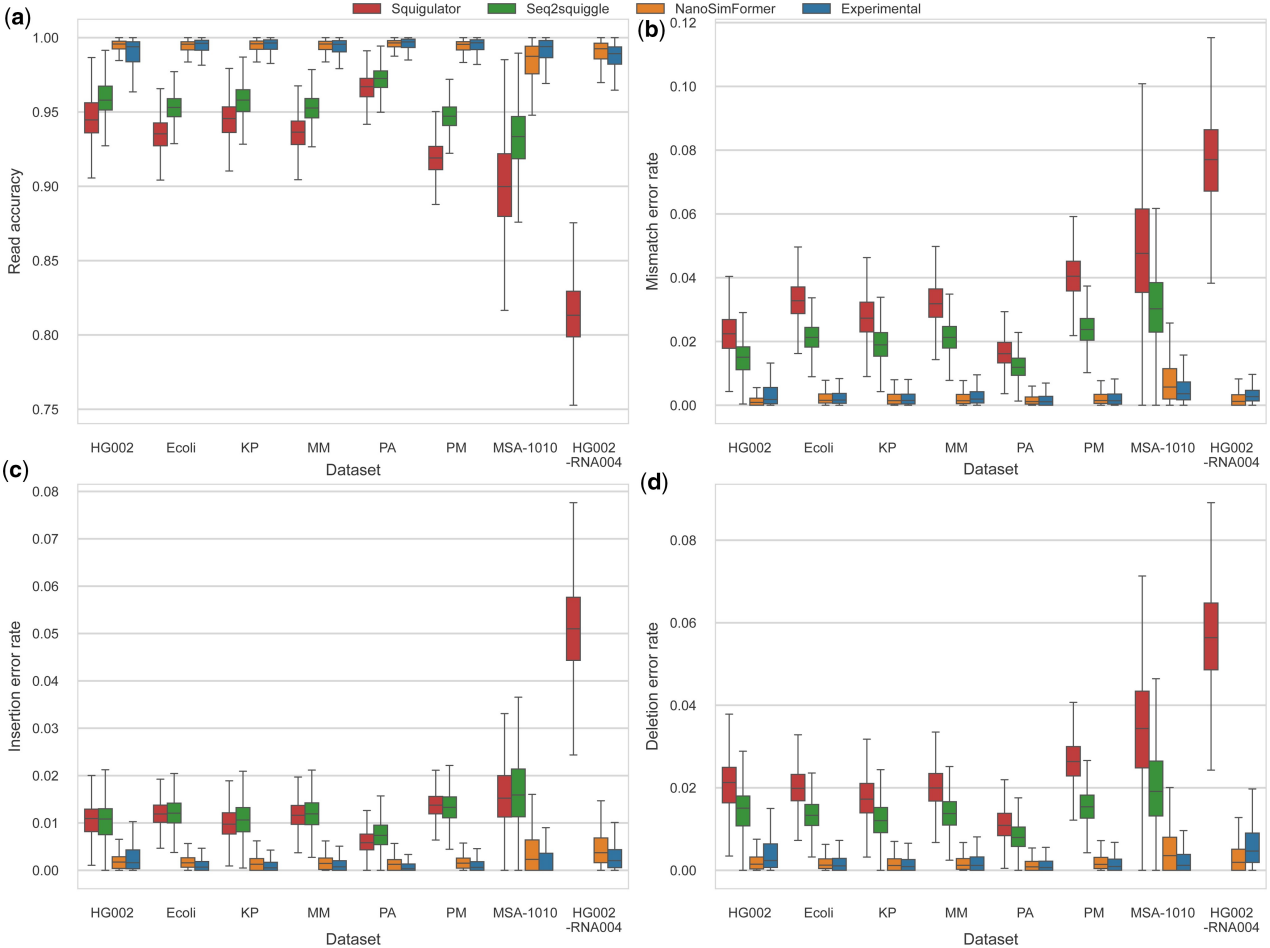
Basecalling performance of different simulators on various species. Performance metrics were evaluated for real experimental data (blue) and simulated signals from NanoSimFormer (orange), seq2squiggle (green), and Squigulator (red) across different datasets. The evaluation metrics include: (a) Read accuracy; (b) Mismatch error rate; (c) Insertion error rate; and (d) Deletion error rate. In the box plots, the center line represents the median, the edges of the box denote the first and third quartiles, thus enclosing the interquartile range (IQR), and the whiskers extend to the most extreme data points within 1.5× IQR from the edges of the box. Sample reads used to derive these statistics are: HG002 (n=320,000), *E. coli* (n=254,210), KP (n=365,393), MM (n=261,589), PA (n=211,667), PM (n=140,727), MSA-1010 (n=1,884,844) and HG002-RNA004 (n=528,128).

In DNA sequencing simulation (Fig. 2; Fig. S2 and Table S5, available as supplementary data at *Bioinformatics* online), NanoSimFormer consistently generated signals with high fidelity, achieving median read accuracies ranging from 98.74% to 99.65%, closely matching the real experimental range of 99.39% to 99.72% (Fig. 2a). In contrast, the competing simulators showed deviations from real experimental data, with seq2squiggle and Squigulator yielding lower median accuracies, ranging from 93.34% to 97.25% and from 89.98% to 96.70%, respectively. This superiority in read accuracy is further evidenced by the PHRED quality scores (Fig. S2a, available as supplementary data at *Bioinformatics* online). NanoSimFormer consistently achieved median Q-scores between 23.3 and 24.7 (indicating >99% base confidence), whereas seq2squiggle and Squigulator produced signals with markedly lower quality, consistently falling below a median Q-score of 16 across all datasets. NanoSimFormer maintained low average mismatch (0.57%), insertion (0.28%), and deletion (0.42%) rates that were close to the experimental baselines (0.48%, 0.27%, and 0.40%, respectively), whereas the other simulators frequently exhibited error rates exceeding 1.0% across these categories (Fig. 2b–d). NanoSimFormer also demonstrated robust generalization capabilities across both human and non-human datasets, whereas the performance of other simulators degraded substantially on non-human data. For instance, on the *Proteus mirabilis* (PM) dataset, NanoSimFormer achieved a median accuracy of 99.55%, closely matching the experimental accuracy of 99.65% and significantly outperforming seq2squiggle (94.72%) and Squigulator (91.91%). In the complex fungal metagenomic dataset (MSA-1010), all simulators exhibited reduced accuracy relative to reference-based simulations, as expected, because the simulation inputs were experimental basecalled reads that inherently contain sequencing errors that propagate into the generated signals. Despite this challenge, NanoSimFormer maintained a high median accuracy of 98.74%, remaining comparable to the experimental 99.40%. The observed accuracy difference (0.66%) is close to the intrinsic experimental read error (0.60%), demonstrating that NanoSimFormer faithfully preserves the sequence identity of the input reads. In contrast, seq2squiggle and Squigulator showed substantial performance degradation, with median accuracies of only 93.34% and 89.98%, respectively. While all simulators produced read length distributions broadly consistent with the real experimental data, NanoSimFormer exhibited the closest overall agreement (Fig. S2b, available as supplementary data at *Bioinformatics* online). NanoSimFormer also demonstrated superior performance on the HG002-RNA004 DRS dataset, achieving a median read accuracy of 99.26% while maintaining error profiles consistent with the experimental baseline. In contrast, Squigulator showed substantially lower performance on RNA004 simulation, with a median accuracy of only 81.33% and markedly higher error rates, exceeding 5% for both insertions and deletions. For signal-level fidelity evaluation using DTW distance, NanoSimFormer consistently achieved the lowest DTW distance across all five independent human datasets (mean: 3.98–6.75; median: 3.86–6.28), outperforming seq2squiggle (mean: 4.86–7.36; median: 4.59–6.76) and Squigulator (mean: 4.33–7.16; median: 4.26–6.33) (Fig. S3 and Table S6, available as supplementary data at *Bioinformatics* online). These results demonstrate that NanoSimFormer more accurately captures the complex temporal dynamics of real nanopore currents and that this advantage in signal-level fidelity generalizes robustly to experimental conditions unseen in training. Furthermore, although NanoSimFormer was trained under the guidance of the Dorado super-accuracy basecalling model (v5.0.0), it demonstrated strong robustness across different basecallers and basecalling configurations (Fig. S4, available as supplementary data at *Bioinformatics* online). When evaluated using multiple R10.4.1 basecalling models of Dorado (v4.2.0, v4.3.0, v5.0.0, and the latest v5.2.0) as well as Guppy under various modes (fast, high, and super accuracy), NanoSimFormer consistently produced read accuracies that closely matched the experimental baselines. This forward compatibility demonstrates that the signal characteristics learned through basecaller-guided training generalize to future basecaller updates without requiring re-training. Collectively, these results demonstrate the high fidelity, robustness, and generalization capability of NanoSimFormer in simulating signals from new nanopore chemistries.

### 3.2 Variant calling performance

**Figure 3:**
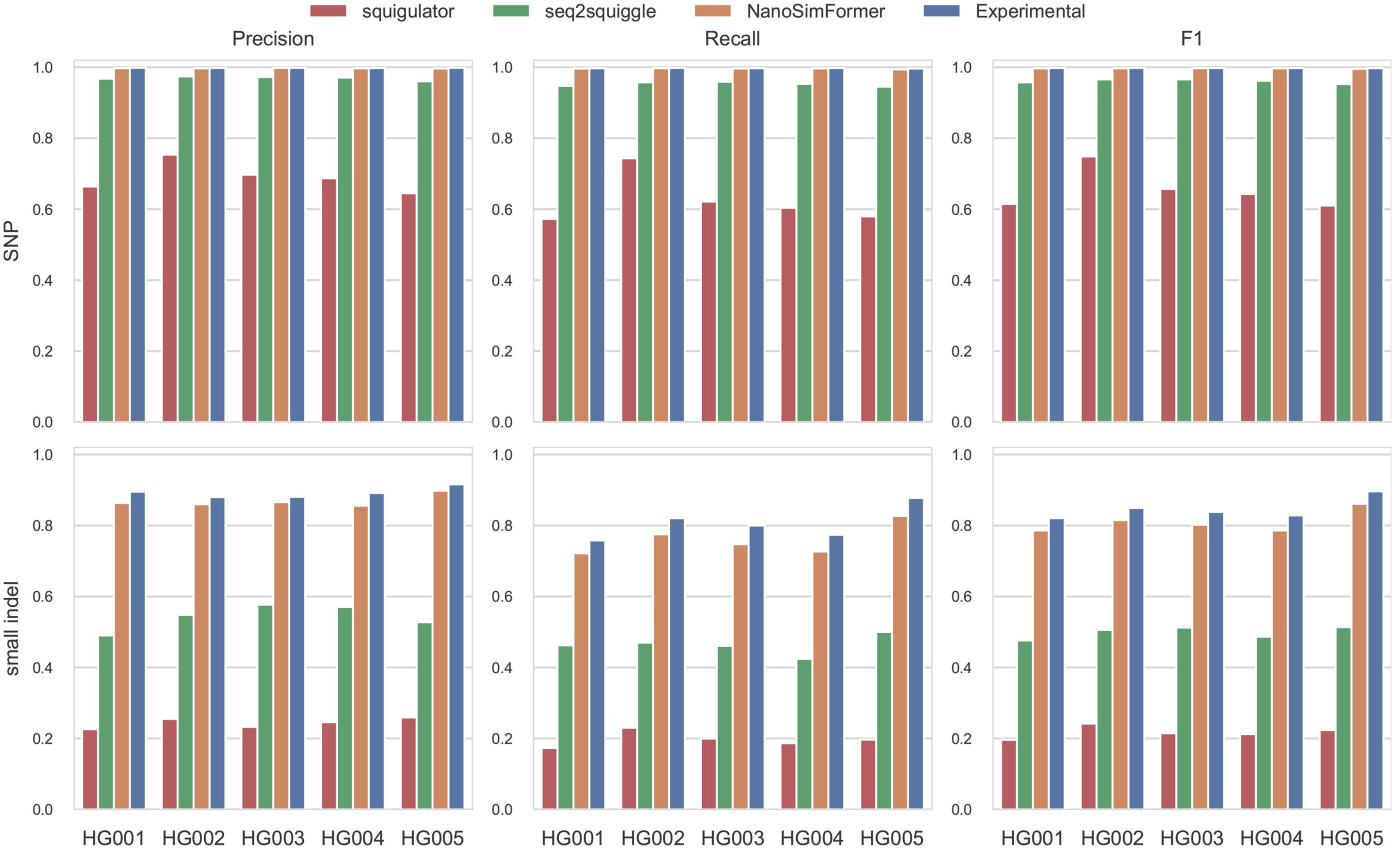
Small variant (SNP and small indel) detection performance across five human DNA R10.4.1 samples on the chromosome 22 reference. Bar plots showing the precision, recall, and F1 score of small variant detection performance under the variant quality threshold yielding the highest F1 score. Results were derived from the human HG001, HG002, HG003, HG004, and HG005 datasets, with sample sizes of n=72,821, n=320,000, n=117,800, n=125,571, and n=77,593, respectively.

NanoSimFormer demonstrated exceptional and consistent precision in SNP detection across five human DNA R10.4.1 datasets (Fig. 3, Table S7, available as supplementary data at *Bioinformatics* online), achieving F1-scores ranging from 0.9953 to 0.9973, closely matching the performance of the real experimental data (0.9974–0.9979). In contrast, seq2squiggle attained moderate F1-scores of 0.9527–0.9658 across datasets, whereas Squigulator showed substantial performance degradation with F1-scores of only 0.6104–0.7486. Performance disparities were further pronounced in the detection of small indels, a challenging modality for nanopore sequencing (Fig. 3, Table S8, available as supplementary data at *Bioinformatics* online). NanoSimFormer maintained high indel F1-scores ranging from 0.7862 to 0.8612 across all five datasets, comparable to the experimental baselines (0.8209–0.8963). Conversely, seq2squiggle and Squigulator struggled to support accurate indel calling, yielding F1-scores of 0.4761–0.5135 and 0.1960–0.2419, respectively. The variant calling results on the HG002 dataset were further stratified by genomic context, with a particular focus on homopolymer and STR regions. Both seq2squiggle and Squigulator produced a high frequency of false-positive (FP) calls within homopolymer regions, whereas NanoSimFormer maintained a low FP rate comparable to that observed in real experimental data (Fig. S5a, available as supplementary data at *Bioinformatics* online). These findings are further supported by the homopolymer-specific error profiles (Fig. S5b–d, available as supplementary data at *Bioinformatics* online), which quantify the insertion, deletion, and mismatch rates across homopolymers of varying lengths. NanoSimFormer consistently exhibited low error rates that closely tracked the real experimental data across all homopolymer lengths. In contrast, seq2squiggle and Squigulator showed markedly elevated error rates—particularly deletion errors—that increased with homopolymer length (Fig. S5c, available as supplementary data at *Bioinformatics* online). These substantial deviations from real experimental error patterns in homopolymer regions likely lead to spurious indel calls during downstream analysis, thereby substantially limiting the suitability of seq2squiggle and Squigulator for robust variant-calling benchmarking. NanoSimFormer also generated simulated signals that enabled highly accurate variant detection for RNA004 DRS, achieving F1-scores of 0.92 and 0.57 for SNPs and small indels detection respectively, closely matching real experimental performance (Fig. S6, available as supplementary data at *Bioinformatics* online). Conversely, Squigulator failed to support meaningful variant calling in RNA004 DRS simulation, yielding negligible F1-scores for small variants (<0.1), primarily due to the low basecalling accuracy of its simulated signals. For structural variant detection, NanoSimFormer achieved an F1-score of 0.8445, comparable to the real experimental data (0.8389) and outperforming both seq2squiggle (0.7918) and Squigulator (0.7970) (Table S9, available as supplementary data at *Bioinformatics* online). Collectively, these findings demonstrate that NanoSimFormer is currently the sole simulator capable of generating synthetic signal datasets suitable for rigorous benchmarking in high-precision variant calling applications.

### 3.3 De novo assembly and structure preservation

**Figure 4:**
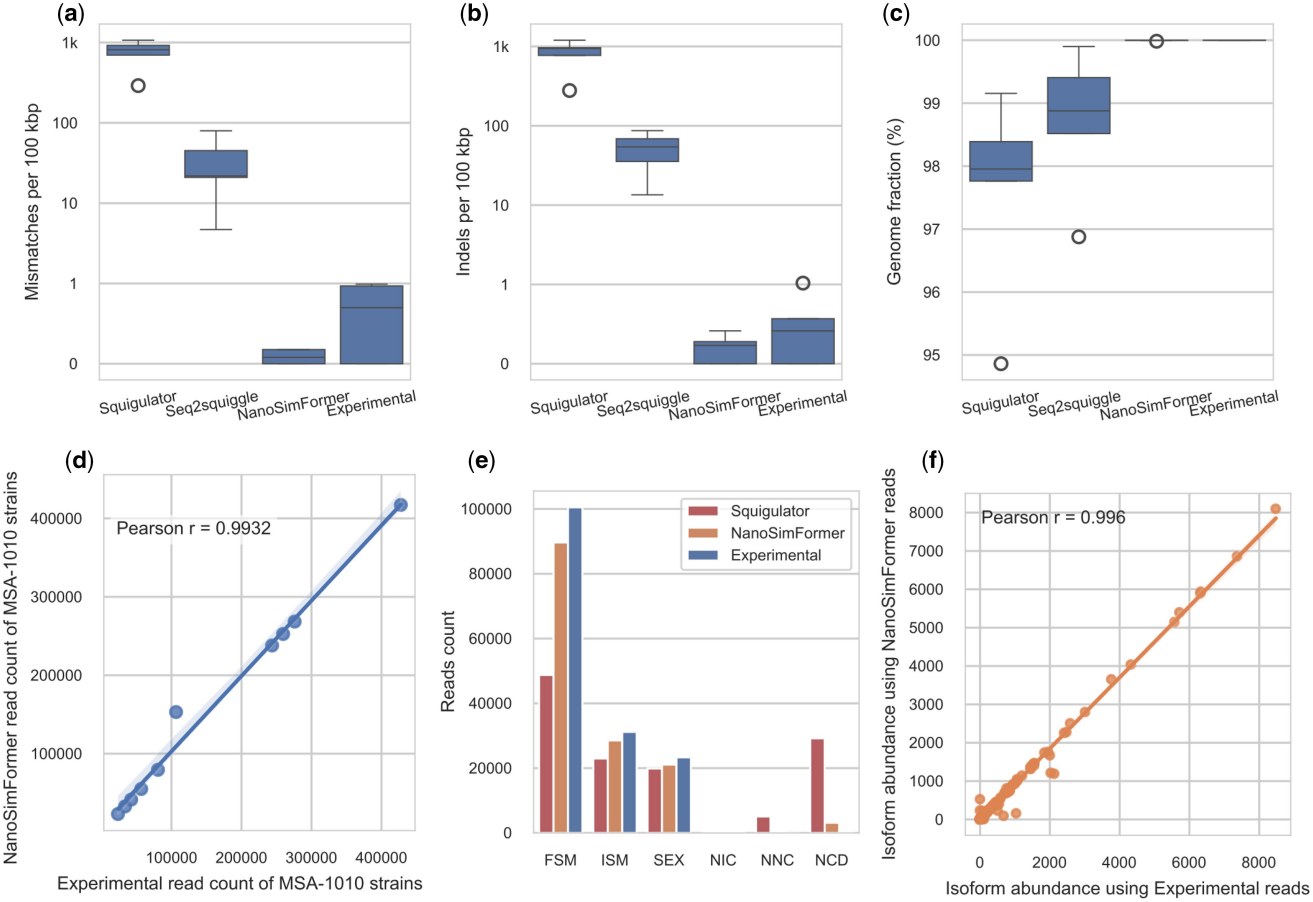
Assessment of de novo genome assembly quality and abundance preservation in simulated data. (a–c) De novo assembly performance was evaluated using simulated and real experimental reads on n=5 biologically independent bacterial datasets. The assemblies were assessed for (a) Mismatches per 100 kbp; (b) Indels per 100 kbp; and (c) Genome fraction (%). For all box plots, the center line represents the median, the lower and upper bounds of the box represent the 25th and 75th percentiles, respectively, and the whiskers extend to the minimum and maximum values within 1.5 times the interquartile range, with outliers displayed as individual points. (d) Scatter plots illustrating the correlation of abundance (read counts classified by minimap2) between the real experimental data and the NanoSimFormer simulated outputs for the MSA-1010 fungal mock community, comprising n=10 distinct fungal strains. (e) Bar plots showing the number of reads classified into transcript categories: FSM, ISM, SEX, NIC, NNC, and NCD using ESPRESSO in the HG002-RNA004 dataset (n=528,128 reads). (f) Scatter plots showing the correlation between transcript expression (quantified as raw abundance) estimates derived from simulated reads generated by NanoSimFormer versus those obtained from real experimental reads in the HG002-RNA004 dataset. The analysis was performed on n=462 transcripts for NanoSimFormer. For panels d and f, the solid line represents the linear regression fit, and the shaded band indicates the 95% confidence interval (CI) of the regression estimate. The Pearson correlation coefficient (*r*) was provided for each comparison.

NanoSimFormer produced assemblies with consensus quality comparable to that obtained from the real experimental data. At the base level, assemblies generated from NanoSimFormer and real experimental reads consistently achieved near-perfect accuracy, with fewer than one mismatch or indel per 100 kbp across all evaluated datasets (Fig. 4a-b). In contrast, assemblies derived from seq2squiggle and Squigulator suffer from mismatch and indel densities ranging from tens to over a thousand per 100 kbp (e.g. Squigulator reached 1,067 mismatches per 100 kbp for *E. coli*). This discrepancy was further reflected in genome fraction coverage (Fig. 4c): NanoSimFormer assemblies achieved 99.98%–100.00% coverage, closely matching the real experimental results (99.99%–100.00%), whereas seq2squiggle and Squigulator frequently produced incomplete assemblies with lower and more variable coverage (e.g. 94.8% for Squigulator on *P. mirabilis*).

On the MSA-1010 fungal metagenomic dataset, NanoSimFormer showed the strongest agreement with real experimental data in community structure preservation, achieving a Pearson correlation coefficient of 0.9932 (Fig. 4d). This indicates that NanoSimFormer faithfully preserves the relative abundance and sequence identity of the input reads in complex metagenomic scenarios. In comparison, seq2squiggle and Squigulator showed lower correlations (0.9577 and 0.9378, respectively; Fig. S7a–b, available as supplementary data at *Bioinformatics* online), suggesting greater deviation from the real experimental profile during the simulation. For transcriptomics simulation, the distribution of ESPRESSO read categories generated by NanoSimFormer closely mirrored the real experimental profile, maintaining a high proportion of FSM reads (Fig. 4e). In contrast, Squigulator produced a distinct distribution with lower FSM reads and an inflated count of NNC/NCD reads. The simulated reads derived from NanoSimFormer showed a near-perfect correlation on isoform expression with those from real experimental data, achieving a Pearson correlation coefficient of 0.996 (Fig. 4f), while Squigulator exhibited a greater variance, particularly at lower expression levels (Fig. S7c, available as supplementary data at *Bioinformatics* online). Collectively, these findings indicate that NanoSimFormer faithfully retains both the relative abundance of complex communities and the structural integrity of transcript isoforms, establishing it as a robust tool for benchmarking metagenomic and transcriptomic analysis pipelines.

### 3.4 Impact of key simulation parameters

NanoSimFormer is featured by two tunable parameters, namely amplitude noise and event duration variance, to enable flexible generation of synthetic datasets with controllable accuracy for diverse benchmarking scenarios. For amplitude noise, NanoSimFormer demonstrated high stability within the standard deviation ($\sigma_{\text{amp}}$) range of 0 to 2.0 for DNA R10.4.1 simulation. The read accuracy remained consistently high in this interval but exhibited a sharp decline as $\sigma_{\text{amp}}$ exceeded 3.0 (Fig. S8a, available as supplementary data at *Bioinformatics* online). This trend was mirrored in variant detection performance. SNP detection maintained a high F1-score of ∼0.997 for $\sigma_{\text{amp}}$ values ≤ 2.0, but dropped progressively to 0.992 at $\sigma_{\text{amp}}=3.0$ and to 0.7031 at $\sigma_{\text{amp}}=4.0$ (Table S10, Figs S8c and S9a–b, available as supplementary data at *Bioinformatics* online). A similar trend was also observed on the small indels detection (Table S11, Figs S8d and S9c–d, available as supplementary data at *Bioinformatics* online) and the DRS RNA004 simulated results (Supplementary Tables S14 and S15, Figs S11a and S11c-d, available as supplementary data at *Bioinformatics* online). In the default setting of simulation, $\sigma_{\text{amp}}$ is randomly sampled per read from a Gamma distribution, which assigns a lower noise level to the majority of reads while subjecting a smaller proportion to higher noise levels (Fig. S12, available as supplementary data at *Bioinformatics* online). This produces a right-skewed distribution of read accuracies that is qualitatively consistent with experimental observations (Fig. S13, available as supplementary data at *Bioinformatics* online). Without this read-level amplitude noise, the model would produce its highest accuracy on reads whose noise characteristics closely match the high-quality signals encountered during training.

In contrast, variations in event duration exerted a markedly weaker and more uniform impact on performance metrics. The scale parameter of the Gamma distribution used in the duration sampler was varied from 0.5 to 6 while keeping the mean fixed, yielding duration standard deviations ranging from 2.45 to 8.49 for DNA R10.4.1 and from 3.87 to 13.42 for DRS RNA004, respectively. For DNA R10.4.1, read accuracy remained remarkably robust across the entire tested range of duration standard deviations, showing minimal fluctuation (Fig. S8b, available as supplementary data at *Bioinformatics* online). Similarly, SNP and indel detection were insensitive to duration variance, maintaining F1-scores at 0.9972–0.9975 and 0.8084–0.8244, respectively (Supplementary Tables S12 and S13, Figs S8e–f and S10, available as supplementary data at *Bioinformatics* online). A similar trend was also observed for DRS RNA004 simulations (Supplementary Tables S16 and S17, Figs S11b and S11e–f, available as supplementary data at *Bioinformatics* online). Consequently, a moderate duration standard deviation of $\sigma_{\text{dur}}=6.0$ and $\sigma_{\text{dur}}=7.75$ is recommended as the default setting for DNA R10.4.1 and DRS RNA004 simulations, respectively, to ensure an optimal balance between SNP and indel detection performance.

### 3.5 Ablation study

To isolate the contribution of basecaller-guided training, we additionally performed an ablation study. The ablated model that uses the identical architecture as NanoSimFormer was trained without the CTC loss (i.e., only event-level mean and noise losses, functionally equivalent to seq2squiggle’s training objective) on the same training dataset. As shown in Fig. S14, available as supplementary data at *Bioinformatics* online, the ablated model exhibits substantially degraded basecalling performance across all evaluation datasets, achieving median read accuracies of 93.18–97.69%, which are comparable to those of seq2squiggle (93.34–97.25%). In contrast, the full NanoSimFormer model consistently achieved performance closely matching the real experimental data. These findings demonstrate that the basecaller-guided training is the primary driver of NanoSimFormer’s superior signal fidelity, and the Transformer architecture alone without basecaller feedback is insufficient to bridge the gap between event-level signal reconstruction accuracy and downstream basecalling performance.

### 3.6 Experimental environment and runtime analysis

All experiments in this study, including model training and simulation benchmarking, were conducted on a Linux server equipped with one 48-core Intel^®^ Xeon^®^ PLATINUM 8558 CPU (2.1 GHz), one NVIDIA H100 GPU, and 128 GB of system RAM. The NanoSimFormer DNA R10.4.1 model was trained on one NVIDIA H100 GPU with a batch size of 128 using mixed-precision training for approximately 104 k steps (∼28 hours, 52 GB peak memory). The RNA004 model was trained under the same hardware configuration with a batch size of 64 for approximately 78k steps (∼20 hours, 43 GB peak memory). Complete training hyperparameters for both models are listed in Table S1, available as supplementary data at *Bioinformatics* online. For simulation inference, we benchmarked NanoSimFormer against seq2squiggle and Squigulator across all evaluation datasets (Table S18). NanoSimFormer achieved throughput of 0.39–0.55 Mbp/s, consistently outperforming seq2squiggle (0.28–0.39 Mbp/s) while requiring comparable or lower peak memory usage (2.33–4.05 GB vs. 3.34–6.55 GB). As expected, Squigulator achieved the highest throughput (1.85–4.57 Mbp/s) with minimal memory footprint (0.16–2.21 GB), reflecting its use of static k-mer pore models rather than neural network inference. While Squigulator is faster and suitable for high-throughput applications with coarse approximations, NanoSimFormer’s deep neural network approach is essential for precise benchmarking. To reduce computational overhead and enable large-scale simulations, NanoSimFormer supports multi-GPU inference, allowing users to distribute the computational workload across multiple devices.

## 4 Discussion

Nanopore sequencing has transformed genomics by enabling analysis of long nucleic acid molecules, but developing accurate analysis algorithms relies on high-fidelity simulators that replicate the complex dynamics of modern flow cell chemistries. While recent flow cells like R10.4.1 achieve read accuracies over 99%, current simulators often rely on static pore models or separate signal generation from basecalling. To address this, we developed NanoSimFormer, an end-to-end Transformer-based simulator that integrates basecaller guidance to generate signals optimized for accurate decoding. Through rigorous evaluation on diverse datasets, we demonstrate that NanoSimFormer outperforms existing simulators and closely mirrors experimental performance across variant calling and assembly tasks.

NanoSimFormer’s flexible architecture can adapt to new sequencing protocols and biotechnological applications. While optimized for DNA R10.4.1 and DRS RNA004 chemistry, its basecaller-guided training can be extended to modification-aware signal simulation, addressing the lack of epitranscriptomics simulators. By incorporating modification-aware basecallers (e.g. Dorado (Cruciani and Novoa 2026), $m^{6}A$Basecaller (Cruciani *et al*. 2025)), NanoSimFormer could generate signals preserving patterns for RNA modifications like N6-methyladenosine (m6A), pseudouridine (ψ), and 5-methylcytosine (m5C) (Luo *et al*. 2026), overcoming the ground-truth bottleneck in modification detection (Katopodi *et al*. 2025).

Beyond genomic benchmarking, NanoSimFormer’s high signal fidelity enables applications in synthetic DNA, molecular tagging, and DNA storage (Meiser *et al*. 2022). As signal-based demultiplexing and barcode design advance via methods like Porcupine (Doroschak *et al*. 2020), TDFPS-Designer (Qi *et al*. 2024), WarpDemuX (van der Toorn *et al*. 2025), and SUSTag (Li *et al*. 2025), NanoSimFormer can help design high-capacity barcode sets, validate signal separability, and minimize crosstalk before synthesis. It can also generate synthesis training data for deep learning classifiers (Doroschak *et al*. 2020, Li *et al*. 2025) for real-time demultiplexing, reducing the need for experimental training libraries. In DNA storage, NanoSimFormer can serve as a critical digital channel model, generating synthetic datasets to replicate indel-dominant error profiles and noise patterns, aiding error-correction codec testing (Ding *et al*. 2025).

## Supplementary Material

btag402_Supplementary_Data

## Data Availability

The sequencing data used in this study are available in the SRA/ENA databases and AWS Open Data registry under the accession codes and links listed in Supplementary Tables S2 and S3. The source code is available at https://github.com/BioinfoSZU/NanoSimFormer.

## References

[btag402-B1] Benoit G , James R, Raguideau S et al High-quality metagenome assembly from nanopore reads with nanoMDBG. Nat Commun 2026;17:3556. 41792155 10.1038/s41467-026-69760-y PMC13087279

[btag402-B2] Beslic D , Kucklick M, Engelmann S et al End-to-end simulation of nanopore sequencing signals with feed-forward transformers. Bioinformatics 2024;41:btae744. 39710838 10.1093/bioinformatics/btae744 PMC11729726

[btag402-B3] Chen Y , Davidson NM, Wan YK et al A systematic benchmark of nanopore long-read RNA sequencing for transcript-level analysis in human cell lines. Nat Methods 2025:22:801–12. 40082608 10.1038/s41592-025-02623-4 PMC11978509

[btag402-B4] Chen W , Zhang P, Song L et al Simulation of nanopore sequencing signals based on BiGRU. Sensors 2020;20:7244. 33348876 10.3390/s20247244 PMC7766754

[btag402-B5] Cleary JG , Braithwaite R, Gaastra K et al Comparing variant call files for performance benchmarking of next-generation sequencing variant calling pipelines. *BioRxiv*, 2015.

[btag402-B6] Cruciani S , Delgado-Tejedor A, Pryszcz LP et al De novo basecalling of RNA modifications at single molecule and nucleotide resolution. Genome Biol 2025;26:38. 40001217 10.1186/s13059-025-03498-6 PMC11853310

[btag402-B7] Cruciani S , Novoa EM. The new era of single-molecule RNA modification detection through nanopore base-calling models. Nat Rev Mol Cell Biol 2026;27:10–8. 41083713 10.1038/s41580-025-00896-3

[btag402-B8] De Coster W , De Rijk P, De Roeck A et al Structural variants identified by oxford nanopore PromethION sequencing of the human genome. Genome Res 2019;29:1178–87. 31186302 10.1101/gr.244939.118 PMC6633254

[btag402-B9] Deamer D , Akeson M, Branton D. Three decades of nanopore sequencing. Nat Biotechnol 2016;34:518–24. 27153285 10.1038/nbt.3423 PMC6733523

[btag402-B10] Ding L , Wang K, Zhang H et al Polus: a transformer-based soft-decision codec enhancement platform for DNA Storage. *Biorxiv*, 2025.

[btag402-B11] Dorey A , Howorka S. Nanopore DNA sequencing technologies and their applications towards single-molecule proteomics. Nat Chem 2024;16:314–34. 38448507 10.1038/s41557-023-01322-x

[btag402-B12] Doroschak K , Zhang K, Queen M et al Rapid and robust assembly and decoding of molecular tags with DNA-based nanopore signatures. Nat Commun 2020;11:5454. 33144581 10.1038/s41467-020-19151-8 PMC7642340

[btag402-B13] English AC , Menon VK, Gibbs RA et al Truvari: refined structural variant comparison preserves allelic diversity. Genome Biol 2022;23:271. 36575487 10.1186/s13059-022-02840-6 PMC9793516

[btag402-B14] Escalona M , Rocha S, Posada D. A comparison of tools for the simulation of genomic next-generation sequencing data. Nat Rev Genet 2016;17:459–69. 27320129 10.1038/nrg.2016.57 PMC5224698

[btag402-B15] Gamaarachchi H , Ferguson JM, Samarakoon H et al Simulation of nanopore sequencing signal data with tunable parameters. Genome Res 2024;34:778–83. 38692839 10.1101/gr.278730.123 PMC11216307

[btag402-B16] Gao Y , Wang F, Wang R et al ESPRESSO: robust discovery and quantification of transcript isoforms from error-prone long-read RNA-seq data. Sci Adv 2023;9:eabq5072. 36662851 10.1126/sciadv.abq5072 PMC9858503

[btag402-B17] Giorgino T. Computing and visualizing dynamic time warping alignments in R: the dtw package. J Stat Soft 2009;31:1–24.

[btag402-B18] Graves A , Fernandez S, Gomez F et al Connectionist temporal classification: labelling unsegmented sequence data with recurrent neural networks. In: *Proceedings of the 23rd International Conference on Machine Learning*, 2006, p. 369–376.

[btag402-B19] Gurevich A , Saveliev V, Vyahhi N et al QUAST: quality assessment tool for genome assemblies. Bioinformatics 2013;29:1072–5. 23422339 10.1093/bioinformatics/btt086 PMC3624806

[btag402-B20] Hafezqorani S , Yang C, Lo T et al Trans-NanoSim characterizes and simulates nanopore RNA-sequencing data. Gigascience 2020;9:giaa061. 32520350 10.1093/gigascience/giaa061 PMC7285873

[btag402-B21] Hansen NF , Dwarshuis N, Ji HJ et al A complete diploid human genome benchmark for personalized genomics. *BioRxiv*, 2025. 10.1016/j.cell.2026.06.016 PMC13456413 42561913

[btag402-B22] Hewel C , Wierczeiko A, Miedema J et al Direct RNA sequencing enables improved transcriptome assessment and tracking of RNA modifications for medical applications. Nucleic Acids Res 2025;53:gkaf1314. 41325774 10.1093/nar/gkaf1314 PMC12663090

[btag402-B23] Jain M , Koren S, Miga KH et al Nanopore sequencing and assembly of a human genome with ultra-long reads. Nat Biotechnol 2018;36:338–45. 29431738 10.1038/nbt.4060 PMC5889714

[btag402-B24] Katopodi X-L , Begik O, Novoa EM. Toward the use of nanopore RNA sequencing technologies in the clinic: challenges and opportunities. Nucleic Acids Res 2025;53:gkaf128. 40057374 10.1093/nar/gkaf128 PMC11890063

[btag402-B25] Li H. Minimap2: pairwise alignment for nucleotide sequences. Bioinformatics 2018;34:3094–100. 29750242 10.1093/bioinformatics/bty191 PMC6137996

[btag402-B26] Li Y , Han R, Bi C et al DeepSimulator: a deep simulator for Nanopore sequencing. Bioinformatics 2018;34:2899–908. 29659695 10.1093/bioinformatics/bty223 PMC6129308

[btag402-B27] Li Y , Wang S, Bi C et al DeepSimulator1.5: a more powerful, quicker and lighter simulator for Nanopore sequencing. Bioinformatics 2020;36:2578–80. 31913436 10.1093/bioinformatics/btz963 PMC7178411

[btag402-B28] Li J , Zhao X, Fan Q et al Empowering low-crosstalk, dynamic-decision random access of DNA storage via 384-multiplexed nanopore signatures. Nat Commun 2025;16:9233. 41107252 10.1038/s41467-025-64293-2 PMC12534545

[btag402-B29] Loose M , Malla S, Stout M. Real-time selective sequencing using nanopore technology. Nat Methods 2016;13:751–4. 27454285 10.1038/nmeth.3930 PMC5008457

[btag402-B30] Luo T , Xu M, Wang M et al Systematic evaluation of computational tools for multitype RNA modification detection using nanopore direct RNA sequencing. Nat Methods 2026;23:438–50. 41372661 10.1038/s41592-025-02974-y

[btag402-B31] Massaiu I , Valerio V, Rusconi V et al Accurate and rapid single nucleotide variation detection in PCSK9 gene using nanopore sequencing. Front Med (Lausanne) 2025;12:1620405. 40933564 10.3389/fmed.2025.1620405 PMC12417156

[btag402-B32] Meiser LC , Nguyen BH, Chen Y-J et al Synthetic DNA applications in information technology. Nat Commun 2022;13:352. 35039502 10.1038/s41467-021-27846-9 PMC8763860

[btag402-B33] Prior K et al Accurate and reproducible whole-genome genotyping for bacterial genomic surveillance with Nanopore sequencing data. J Clin Microbiol 2025;63:e00369 25. 40511924 10.1128/jcm.00369-25 PMC12239720

[btag402-B34] Qi J , Li Z, Zhang Y-Z et al TDFPS-Designer: an efficient toolkit for barcode design and selection in nanopore sequencing. Genome Biol 2024;25:285. 39497190 10.1186/s13059-024-03423-3 PMC11533379

[btag402-B35] Shafin K , Pesout T, Chang P-C et al Haplotype-aware variant calling with PEPPER-Margin-DeepVariant enables high accuracy in nanopore long-reads. Nat Methods 2021;18:1322–32. 34725481 10.1038/s41592-021-01299-w PMC8571015

[btag402-B36] Shazeer N. Glu variants improve transformer. *arXiv preprint, arXiv : 2002.05202.* 2020, preprint: not peer reviewed.

[btag402-B37] Simpson JT , Workman RE, Zuzarte PC et al Detecting DNA cytosine methylation using nanopore sequencing. Nat Methods 2017;14:407–10. 28218898 10.1038/nmeth.4184

[btag402-B38] Smolka M , Paulin LF, Grochowski CM et al Detection of mosaic and population-level structural variants with Sniffles2. Nat Biotechnol 2024;42:1571–80. 38168980 10.1038/s41587-023-02024-y PMC11217151

[btag402-B39] Su J , Ahmed M, Lu Y et al Roformer: enhanced transformer with rotary position embedding. Neurocomputing (Amst) 2024;568:127063.

[btag402-B40] van der Toorn W , Bohn P, Liu-Wei W et al Demultiplexing and barcode-specific adaptive sampling for nanopore direct RNA sequencing. Nat Commun 2025;16:3742. 40258808 10.1038/s41467-025-59102-9 PMC12012114

[btag402-B41] Wick RR. Badread: simulation of error-prone long reads. JOSS 2019;4:1316.

[btag402-B42] Wong B , Ferguson JM, Do JY et al Streamlining remote nanopore data access with slow5curl. GigaScience 2024;13:giae016. 38608279 10.1093/gigascience/giae016 PMC11010652

[btag402-B43] Yang C , Chu J, Warren RL et al NanoSim: nanopore sequence read simulator based on statistical characterization. Gigascience 2017;6:gix010. 10.1093/gigascience/gix010 PMC5530317 28327957

[btag402-B44] Zheng Z , Li S, Su J et al Symphonizing pileup and full-alignment for deep learning-based long-read variant calling. Nat Comput Sci 2022;2:797–803. 38177392 10.1038/s43588-022-00387-x

[btag402-B45] Zheng Z , Yu X, Chen L et al Clair3-RNA: a deep learning-based small variant caller for long-read RNA sequencing data. Nat Commun 2025;16:11553. 10.1038/s41467-025-67237-y 41430046 PMC12748580

[btag402-B46] Zook JM , Catoe D, McDaniel J et al Extensive sequencing of seven human genomes to characterize benchmark reference materials. Sci Data 2016;3:160025–6. 27271295 10.1038/sdata.2016.25 PMC4896128

