# Supplementary material for: NanoSimFormer: an end-to-end transformer-based nanopore signal simulator with basecaller guidance
Source: Bioinformatics. 2026 Jun 16;42(6):btag402. doi: 10.1093/bioinformatics/btag402 (PMC13312124; doi:10.1093/bioinformatics/btag402)
Supplement: btag402_Supplementary_Data [file btag402_supplementary_data.pdf]

# NanoSimFormer: An end-to-end Transformer-based simulator for nanopore sequencing signal data

## Table of contents

Table of contents .......... 1

Supplementary Note S1 .......... 3

Table S1 .......... 5

Table S2 .......... 6

Table S3 .......... 7

Table S4 .......... 8

Table S5 .......... 9

Table S6 .......... 10

Table S7 .......... 11

Table S8 .......... 12

Table S9 .......... 13

Table S10 .......... 14

Table S11 .......... 15

Table S12 .......... 16

Table S13 .......... 17

Table S14 .......... 18

Table S15 .......... 19

Table S16 .......... 20

Table S17 .......... 21

Table S18 .......... 22

Fig S1 .......... 23

Fig S2 .......... 24

Fig S3 .......... 25

Fig S4 .......... 26

Fig S5 .......... 27

Fig S6 .......... 28

**Fig S7** .......... **29**

**Fig S8** .......... **30**

**Fig S9** .......... **31**

**Fig S10** .......... **32**

**Fig S11** .......... **33**

**Fig S12** .......... **34**

**Fig S13** .......... **35**

**Fig S14** .......... **36**

## Supplementary Note S1. Downstream analysis details

### 1. Basecalling accuracy

All simulated and real experimental raw signals were basecalled using **Dorado (v1.3.0)** with super-accuracy models (`dna_r10.4.1_e8.2_400bps_sup@v5.0.0` for R10.4.1 reads; `rna004_130bps_sup@v5.0.0` for RNA004 reads). After basecalling, reads were aligned using **minimap2 (v2.28-r1209)**. Unaligned reads and supplementary alignments were excluded.

```
$ dorado basecaller --recursive --emit-fastq --device cuda:gpu_id dna_r10.4.1_e8.2_400bps_sup@v5.0.0 sim.pod5 > sim.fastq
$ minimap2 -y -ax lr:hq -t 72 --eqx --MD --secondary=no ref.fasta sim.fastq | samtools view -Sb --threads 72 -o sim_aligned.bam
$ samtools sort sim_aligned.bam -o sim_aligned_sorted.bam && mv sim_aligned_sorted.bam sim_aligned.bam && samtools index sim_aligned.bam
```

### 2. Variant Calling

- **Small variants (SNPs and indels)**: Called using **Clair3 (v1.2.0)** for DNA R10.4.1 reads, or **Clair3-RNA (v0.2.2)** for RNA004 DRS. On DNA reads, the default Clair3 R10.4.1 super-accuracy model (`r1041_e82_400bps_sup_v500`) was used for real experimental data, NanoSimFormer, and seq2squiggle; the `r1041_e82_400bps_sup_v410` model was used for Squigulator as it achieved better results compared to the default selection. VCFs were filtered (QUAL>0 and DP>1) with **bcftools** and benchmarked against **GIAB (v3.3.2)** using **RTG tools (v3.13)**.

```
$ minimap2 -ax lr:hq --secondary=no hg38.fasta sim.fastq | samtools sort -o hg38_aln.bam && samtools index hg38_aln.bam     # For DNA R10.4.1 reads
$ minimap2 -ax splice -uf --secondary=no hg38.fasta sim.fastq | samtools sort -o hg38_aln.bam && samtools index hg38_aln.bam  # For RNA004 reads
$ run_clair3.sh --include_all_ctgs --bam_fn= hg38_aln.bam --ref_fn=hg38.fasta --platform=ont --model_path= r1041_e82_400bps_sup_v500 --output=out_dir --sample_name=reads # For DNA R10.4.1 reads
$ run_clair3_rna --bam_fn hg38_aln.bam --ref_fn hg38.fasta --output_dir out_dir --platform ont_dorado_drna004 --tag_variant_using_readiportal --ctg_name chr22    # For RNA004 reads
$ bcftools filter -i 'QUAL>0 && DP>1' out_dir/merge_output.vcf.gz -Ov -o out_dir/merge_output_filtered.vcf.gz
$ rtg vcfeval -b GIAB_truth.vcf.gz -c out_dir/merge_output_filtered.vcf.gz -t hg38.sdf --region chr22 -e GIAB_bench.bed -f QUAL -o rtg_out
```

- **Structural variants**: Detected using **Sniffles2 (v2.7.0)** with tandem repeat annotations. Evaluated against the GIAB HG002 SV Draft Benchmark (V0.019-20241113, T2T-HG002-Q100v1.1) using **Truvari (v5.4.0)**.

```
$ sniffles -i hg38_aln.bam -v sv_out.vcf --tandem-repeats hg38_trf.bed --
reference hg38.fasta && bgzip sv_out.vcf
$ truvari bench -b GIAB_truth.vcf.gz -c sv_out.vcf.gz -f hg38.fasta -o
truvari_out --pick ac --passonly -r 2000 -C 5000 --includebed GIAB_bench.bed
$ truvari refine --coords R --use-original-vcfs --align mafft --reference
hg38.fasta --regions truvari_out/candidate.refine.bed truvari_out
```

### 3. De Novo Assembly

Bacterial genomes were assembled using **nanoMDBG (v1.2)**, polished with **Medaka (v2.1.1)**, and evaluated using **QUAST (v5.2.0)**.

```
$ metaMDBG asm --in-ont sim.fastq --out-dir asm_out
$ pigz -d asm_out/contigs.fasta.gz
$ medaka_consensus -d asm_out/contigs.fasta -i sim.fastq -o medaka_out
$ quast.py -o quast_out  -r ref.fasta medaka_out/consensus.fasta
```

### 4. Abundance Analysis

Metagenomic community composition was evaluated by aligning basecalled reads to the MSA-1010 composite reference genome with minimap2. Transcript isoform quantification was performed using **ESPRESSO (v1.6.0)**.

```
$ perl ESPRESSO_S.pl -L samples.tsv -F hg38.fasta -O espresso_out -A
hg002_chr22.gtf
$ perl ESPRESSO_C.pl -I espresso_out -F hg38.fasta -X 0 -T 20
$ perl ESPRESSO_Q.pl -L espresso_out/samples.tsv.updated
-V espresso_out/samples_N2_R0_compatible_isoform.tsv -A hg002_chr22.gtf
```

## Table S1. Training setting

| | DNA R10.4.1 Training config | RNA004 Training config |
|---|---|---|
| Batch size | 128 | 64 |
| Signal chunk length | 5000 | 12,000 |
| Maximum nucleotide sequence length (bp) | 500 | 500 |
| Chunk number | 13403827 | 5031242 |
| Step size | 104717 | 78613 |
| Feature dimension | 512 | 512 |
| Attention head number | 8 | 8 |
| Sequence Transformer encoder layers | 12 | 12 |
| Optimizer | AdamW | AdamW |
| Optimizer betas | (0.9, 0.999) | (0.9, 0.999) |
| Optimizer eps | 1e-08 | 1e-08 |
| Optimizer weight decay | 0.01 | 0.01 |
| Initial learning rate | 2e-4 | 2e-4 |
| Learning rate scheduler | Linear Warmup Cosine Annealing | Linear Warmup Cosine Annealing |
| Warmup steps | 500 | 500 |
| Automatic mixed precision | Float16 | Float16 |
| Gradient norm clip | 1.0 | 1.0 |
| Training time (hour) | 28 | 20 |
| Maximum memory usage (GB) | 52 | 43 |

## Table S2. Evaluation dataset

| Dataset | Species | Flow cell | Library kit | Read number | Link or SRA/ENA accession ID | Link or NCBI accession ID of reference |
|---|---|---|---|---|---|---|
| HG002 (chr22) | *Homo sapiens* | FLO-PRO114M | SQK-LSK114 | 320000 | ERR12997168 | https://s3-us-west-2.amazonaws.com/human-pangenomics/T2T/HG002/assemblies/hg002v1.1.fasta.gz |
| E.coli | *Escherichia coli* | FLOMIN-114 | SQK-RBK114 | 254210 | ERR14711428 | https://zenodo.org/records/18220606/files/Ecoli_ref.fasta |
| KP | *Klebsiella pneumoniae* | FLOMIN-114 | SQK-RBK114 | 365393 | ERR14720565 | https://zenodo.org/records/18220606/files/Klebsiella_pneumoniae_ref.fasta |
| MM | *Morganella morganii* | FLOMIN-114 | SQK-RBK114 | 261589 | ERR14720737 | https://zenodo.org/records/18220606/files/Morganella_morganii_ref.fasta |
| PA | *Pseudomonas aeruginosa* | FLOMIN-114 | SQK-RBK114 | 211667 | ERR14720772 | https://zenodo.org/records/18220606/files/Pseudomonas_aeruginosa_ref.fasta |
| PM | *Proteus mirabilis* | FLOMIN-114 | SQK-RBK114 | 140727 | ERR14720750 | https://zenodo.org/records/18220606/files/Proteus_mirabilis_ref.fasta |
| MSA-1010 | Fungal mock community (10 fungal strains) | FLO-MIN114 | SQK-MAB114 | 1884844 | s3://ont-open-data/fungal_ITS_2025.09/raw | ASM265v1, CNA3, ASM1935993v1, ASM71027v1, ASM2543354v1, ASM18296v3, CBS138_2MG, ASM18169v2, ASM4408963v1, ASM2001101v1 |
| HG002-RNA004 (chr22) | *Homo sapiens* | FLO-PRO004RA | SQK-RNA004 | 528128 | SRR30901279 | CP139520.2, CP139543.2 |

## Table S3. Human DNA R10.4.1 datasets for variant calling and DTW benchmark

| **Dataset** | **Flow cell** | **Library kit** | **Read number** | **Link or SRA/ENA accession ID** | **Reference for variant calling** |
|---|---|---|---|---|---|
| HG001 (chr22) | FLO-PRO114M | SQK-LSK114 | 72821 | s3://ont-open-data/giab_2025.01/flowcells/HG001 | GRCh38 |
| HG002 (chr22) | FLO-PRO114M | SQK-LSK114 | 320000 | ERR12997168 | GRCh38 |
| HG003 (chr22) | FLO-PRO114M | SQK-LSK114 | 117800 | s3://ont-open-data/giab_2025.01/flowcells/HG003 | GRCh38 |
| HG004 (chr22) | FLO-PRO114M | SQK-LSK114 | 125571 | s3://ont-open-data/giab_2025.01/flowcells/HG004 | GRCh38 |
| HG005 (chr22) | FLO-PRO114M | SQK-LSK114 | 77593 | s3://ont-open-data/giab_2025.01/flowcells/HG005 | GRCh38 |

## Table S4. Simulator configuration

| Software Name (version) | Download url | Command |
|---|---|---|
| Squigulator (v0.4.0) | https://github.com/hasindu2008/squigulator | squigulator [reference] -x [dna-r10-prom \| rna004-prom] --seed 42 -r [mean_read_length] -o [output_blow5] -n [read_number] --ont-friendly=yes |
| Seq2squiggle (v0.3.4) | https://github.com/ZKI-PH-ImageAnalysis/seq2squiggle | seq2squiggle predict [reference] -o [output_blow5] --preserve-read-ids --num-reads [read_number] --read-length [mean_read_length] --seed 42 --profile dna-r10-prom --model R10_4_1.ckpt |
| NanoSimFormer (v1.1) | https://github.com/BioinfoSZU/NanoSimFormer | python nano_signal_simulator.py --input [reference] --output [output_pod5] --mode Reference --preset [ont_r1041_dna_5khz \| ont_rna004_4khz] --seed 42 --gpu [GPU_ID] --batch-size [batch_size] --sample-reads [read_number] |

## Table S5. Simulation basecalling performance

| Dataset | Method | Read Accuracy (%) | | PHRED Quality Score | | Mismatch (%) | | Insertion(%) | | Deletion (%) | | Read Length (bp) | |
|---|---|---|---|---|---|---|---|---|---|---|---|---|---|
| | | mean | median | mean | median | mean | median | mean | median | mean | median | mean | median |
| HG002 (chr22) | Experimental | 97.816 | 99.388 | 22.475 | 23.458 | 0.825 | 0.181 | 0.546 | **0.161** | 0.813 | 0.238 | 6636 | 2603 |
| | NanoSimFormer | **98.956** | **99.569** | **23.180** | **23.930** | **0.385** | **0.088** | **0.254** | 0.175 | **0.405** | **0.150** | 6666 | 2624 |
| | Seq2squiggle | 96.038 | 95.795 | 15.344 | 14.755 | 1.518 | 1.508 | 1.013 | 1.082 | 1.431 | 1.507 | 6804 | 4785 |
| | Squigulator | 94.773 | 94.467 | 14.02 | 13.491 | 2.239 | 2.243 | 1.020 | 1.093 | 1.969 | 2.128 | 6689 | 5617 |
| E.coli | Experimental | **99.043** | **99.617** | **24.84** | **25.272** | **0.398** | 0.163 | **0.227** | **0.068** | **0.331** | **0.107** | 4394 | 2525 |
| | NanoSimFormer | 98.854 | 99.556 | 22.742 | 23.662 | 0.528 | **0.155** | 0.253 | 0.159 | 0.365 | 0.127 | 4469 | 3137 |
| | Seq2squiggle | 95.263 | 95.303 | 13.871 | 13.835 | 2.152 | 2.129 | 1.223 | 1.207 | 1.362 | 1.333 | 4421 | 3099 |
| | Squigulator | 93.453 | 93.529 | 12.398 | 12.377 | 3.316 | 3.278 | 1.203 | 1.190 | 2.027 | 1.987 | 4347 | 3648 |
| KP | Experimental | **99.192** | **99.641** | **25.32** | **25.513** | **0.344** | 0.152 | **0.186** | **0.054** | **0.279** | **0.090** | 2767 | 1777 |
| | NanoSimFormer | 98.958 | 99.583 | 23.192 | 23.912 | 0.482 | **0.144** | 0.225 | 0.129 | 0.334 | 0.117 | 2851 | 2009 |
| | Seq2squiggle | 95.722 | 95.797 | 14.283 | 14.218 | 1.936 | 1.895 | 1.092 | 1.062 | 1.250 | 1.206 | 2801 | 1972 |
| | Squigulator | 94.402 | 94.558 | 13.271 | 13.299 | 2.808 | 2.732 | 1.008 | 0.975 | 1.782 | 1.725 | 2750 | 2308 |
| MM | Experimental | **99.013** | 99.552 | **24.314** | **24.583** | **0.410** | 0.195 | **0.234** | **0.073** | **0.342** | **0.120** | 3259 | 1833 |
| | NanoSimFormer | 98.904 | **99.573** | 22.882 | 23.694 | 0.499 | **0.142** | 0.239 | 0.145 | 0.358 | 0.123 | 3345 | 2353 |
| | Seq2squiggle | 95.232 | 95.266 | 13.876 | 13.826 | 2.152 | 2.129 | 1.211 | 1.193 | 1.405 | 1.376 | 3291 | 2311 |
| | Squigulator | 93.550 | 93.643 | 12.52 | 12.519 | 3.232 | 3.183 | 1.181 | 1.163 | 2.037 | 1.998 | 3233 | 2712 |
| PA | Experimental | **99.313** | **99.715** | **25.971** | **26.176** | **0.284** | **0.111** | **0.159** | **0.035** | **0.244** | **0.068** | 3356 | 1760 |
| | NanoSimFormer | 99.112 | 99.645 | 24.037 | 24.749 | 0.403 | 0.114 | 0.206 | 0.127 | 0.279 | 0.085 | 3434 | 2418 |
| | Seq2squiggle | 97.130 | 97.251 | 15.746 | 15.737 | 1.247 | 1.189 | 0.775 | 0.737 | 0.849 | 0.798 | 3380 | 2367 |
| | Squigulator | 96.520 | 96.700 | 15.293 | 15.363 | 1.706 | 1.619 | 0.624 | 0.583 | 1.150 | 1.088 | 3342 | 2804 |
| PM | Experimental | **99.089** | **99.652** | **25.351** | **25.7** | **0.380** | **0.143** | **0.220** | **0.062** | **0.312** | **0.092** | 3557 | 2078 |
| | NanoSimFormer | 98.776 | 99.546 | 22.829 | 23.679 | 0.553 | 0.149 | 0.248 | 0.151 | 0.422 | 0.144 | 3633 | 2564 |
| | Seq2squiggle | 94.692 | 94.715 | 13.543 | 13.503 | 2.398 | 2.377 | 1.345 | 1.329 | 1.564 | 1.539 | 3587 | 2515 |
| | Squigulator | 91.893 | 91.908 | 11.603 | 11.571 | 4.065 | 4.046 | 1.384 | 1.376 | 2.658 | 2.637 | 3514 | 2944 |
| MSA-1010 (read mode) | Experimental | **98.499** | **99.395** | 21.9 | 22.141 | **0.724** | **0.360** | **0.322** | **0.000** | **0.456** | **0.120** | 604 | 534 |
| | NanoSimFormer | 97.627 | 98.742 | **22.919** | **23.272** | 1.116 | 0.572 | 0.508 | 0.231 | 0.749 | 0.355 | 609 | 537 |
| | Seq2squiggle | 93.017 | 93.341 | 12.764 | 12.708 | 3.203 | 3.025 | 1.694 | 1.591 | 2.086 | 1.913 | 614 | 535 |
| | Squigulator | 90.016 | 89.981 | 10.988 | 10.903 | 4.902 | 4.762 | 1.595 | 1.524 | 3.487 | 3.436 | 608 | 528 |
| HG002-RNA004 (chr22) | Experimental | 98.281 | 98.924 | **23.27** | 23.379 | **0.373** | 0.271 | **0.531** | **0.205** | 0.816 | 0.467 | 1238 | 1274 |
| | NanoSimFormer | **98.291** | **99.255** | 22.667 | **23.408** | 0.572 | **0.122** | 0.616 | 0.373 | **0.521** | **0.194** | 1175 | 1230 |
| | Squigulator | 81.47 | 81.328 | 9.756 | 9.698 | 7.661 | 7.705 | 5.126 | 5.1 | 5.742 | 5.638 | 1188 | 1196 |

The best results among all methods are marked as bold text.

The best results among simulation tools are underlined.

**Table S6. Normalized Dynamic Time Warping (DTW) distance between simulated and experimental reads for different simulators across human DNA R10.4.1 datasets**

| Dataset | Max raw signal length | Max read length (bp) | Method | Normalized DTW distance | |
|---|---|---|---|---|---|
| | | | | mean | median |
| HG001 | 155418 | 6000 | NanoSimFormer | **4.098** | **4.004** |
| | | | seq2squiggle | 4.913 | 4.716 |
| | | | Squigulator | 4.326 | 4.256 |
| HG002 | 187254 | 6000 | NanoSimFormer | **6.753** | **6.278** |
| | | | seq2squiggle | 7.357 | 6.759 |
| | | | Squigulator | 7.157 | 6.332 |
| HG003 | 188958 | 6000 | NanoSimFormer | **4.117** | **3.932** |
| | | | seq2squiggle | 4.864 | 4.586 |
| | | | Squigulator | 4.438 | 4.27 |
| HG004 | 144396 | 6000 | NanoSimFormer | **3.977** | **3.858** |
| | | | seq2squiggle | 5.39 | 5.239 |
| | | | Squigulator | 4.622 | 4.465 |
| HG005 | 158466 | 6000 | NanoSimFormer | **5.239** | **4.375** |
| | | | seq2squiggle | 6.993 | 6.703 |
| | | | Squigulator | 6.405 | 5.651 |

The best results among all methods are marked as bold text.

All datasets are independent, with 20,000 reads (≤ 6,000 bp) randomly sampled from each dataset for evaluation.

## Table S7. chr22 SNP detection performance of human DNA R10.4.1 samples for different simulators at the best-F1 threshold

| Dataset | Method | Variant quality score threshold | F1 | Precision | Recall | Precision-recall AUC |
|---|---|---|---|---|---|---|
| HG001 | Experimental | 7.24 | **0.9977** | **0.9983** | **0.9970** | **0.9923** |
| | NanoSimFormer | 7.61 | <u>0.9969</u> | <u>0.9974</u> | <u>0.9963</u> | <u>0.9913</u> |
| | seq2squiggle | 17.24 | 0.9574 | 0.9676 | 0.9474 | 0.9669 |
| | Squigulator | 17.32 | 0.6148 | 0.6637 | 0.5726 | 0.6145 |
| HG002 | Experimental | 3.84 | **0.9979** | **0.9977** | **0.9980** | 0.9886 |
| | NanoSimFormer | 4.54 | <u>0.9971</u> | <u>0.9968</u> | <u>0.9973</u> | **<u>0.9935</u>** |
| | seq2squiggle | 17.07 | 0.9657 | 0.9740 | 0.9574 | 0.9685 |
| | Squigulator | 15.32 | 0.7486 | 0.7536 | 0.7436 | 0.7669 |
| HG003 | Experimental | 2.01 | **0.9976** | **0.9981** | **0.9970** | **0.9912** |
| | NanoSimFormer | 5.39 | <u>0.9973</u> | <u>0.9981</u> | <u>0.9965</u> | <u>0.9886</u> |
| | seq2squiggle | 15.83 | 0.9658 | 0.9725 | 0.9592 | 0.9742 |
| | Squigulator | 16.75 | 0.6571 | 0.6970 | 0.6214 | 0.6692 |
| HG004 | Experimental | 4.05 | **0.9976** | **0.9975** | **0.9976** | **0.9918** |
| | NanoSimFormer | 5.81 | <u>0.9970</u> | <u>0.9972</u> | <u>0.9967</u> | <u>0.9876</u> |
| | seq2squiggle | 17.18 | 0.9619 | 0.9709 | 0.9529 | 0.9709 |
| | Squigulator | 17.02 | 0.6429 | 0.6873 | 0.6039 | 0.6536 |
| HG005 | Experimental | 7.97 | **0.9974** | **0.9984** | **0.9963** | **0.9891** |
| | NanoSimFormer | 10.41 | <u>0.9953</u> | <u>0.9965</u> | <u>0.9940</u> | <u>0.9737</u> |
| | seq2squiggle | 17.32 | 0.9527 | 0.9605 | 0.9450 | 0.9620 |
| | Squigulator | 17.70 | 0.6104 | 0.6448 | 0.5795 | 0.6200 |

The best results among all methods are marked as bold text.

The best results among simulation tools are underlined.

**Table S8. chr22 small indels detection performance of human DNA R10.4.1 samples for different simulators at the best-F1 threshold**

| Dataset | Method | Variant quality score threshold | F1 | Precision | Recall | Precision-recall AUC |
|---|---|---|---|---|---|---|
| HG001 | Experimental | 9.30 | **0.8209** | **0.8953** | **0.7579** | **0.7757** |
| | NanoSimFormer | 10.18 | 0.7862 | 0.8636 | 0.7216 | 0.7513 |
| | seq2squiggle | 17.68 | 0.4761 | 0.4904 | 0.4626 | 0.3721 |
| | Squigulator | 18.63 | 0.1960 | 0.2261 | 0.1730 | 0.0977 |
| HG002 | Experimental | 5.78 | **0.8494** | **0.8801** | **0.8207** | **0.8055** |
| | NanoSimFormer | 9.93 | 0.8154 | 0.8599 | 0.7752 | 0.7745 |
| | seq2squiggle | 19.35 | 0.5060 | 0.5482 | 0.4699 | 0.4037 |
| | Squigulator | 18.27 | 0.2419 | 0.2549 | 0.2302 | 0.1458 |
| HG003 | Experimental | 6.27 | **0.8384** | **0.8808** | **0.8000** | **0.7905** |
| | NanoSimFormer | 10.10 | 0.8022 | 0.8660 | 0.7472 | 0.7660 |
| | seq2squiggle | 19.30 | 0.5125 | 0.5769 | 0.4610 | 0.4119 |
| | Squigulator | 18.48 | 0.2147 | 0.2326 | 0.1995 | 0.1158 |
| HG004 | Experimental | 8.12 | **0.8286** | **0.8917** | **0.7737** | **0.7775** |
| | NanoSimFormer | 10.00 | 0.7862 | 0.8559 | 0.7269 | 0.7461 |
| | seq2squiggle | 19.49 | 0.4867 | 0.5706 | 0.4243 | 0.3863 |
| | Squigulator | 18.94 | 0.2122 | 0.2459 | 0.1866 | 0.1085 |
| HG005 | Experimental | 9.41 | **0.8963** | **0.9160** | **0.8775** | **0.8771** |
| | NanoSimFormer | 12.42 | 0.8612 | 0.8983 | 0.8270 | 0.8454 |
| | seq2squiggle | 19.02 | 0.5135 | 0.5276 | 0.5002 | 0.4322 |
| | Squigulator | 19.56 | 0.2238 | 0.2593 | 0.1968 | 0.1244 |

The best results among all methods are marked as bold text.

The best results among simulation tools are underlined.

## Table S9. Performance of structural variant detection on HG002 chr22 dataset

| Methods | TP | FP | FN | Precision | Recall | F1 |
|---|---|---|---|---|---|---|
| Experimental | 384 | **26** | 136 | **0.9366** | 0.7597 | 0.8389 |
| NanoSimFormer | **385** | 27 | **130** | 0.9345 | **0.7703** | **0.8445** |
| Seq2squiggle | 371 | 54 | 156 | 0.8729 | 0.7244 | 0.7918 |
| Squigulator | 362 | 29 | 170 | 0.9258 | 0.6996 | 0.7970 |

The best results among all methods are marked as bold text.

The best results among simulation tools are underlined.

**Table S10. HG002 chr22 SNPs detection performance (under the threshold with the best F1 score) for NanoSimFormer simulated signal using different amplitude noise variance**

| Amplitude noise stdev | Variant quality score threshold | F1 | Precision | Recall | Precision-recall AUC |
|---|---|---|---|---|---|
| 0 | 7.17 | **0.9974** | **0.9974** | 0.9973 | 0.9951 |
| 0.5 | 4.32 | 0.9974 | 0.9972 | **0.9975** | **0.9953** |
| 1 | 4.29 | 0.9972 | 0.9968 | 0.9975 | 0.9951 |
| 2 | 11.04 | 0.9965 | 0.9969 | 0.9961 | 0.9943 |
| 3 | 14.35 | 0.9920 | 0.9929 | 0.9911 | 0.9912 |
| 4 | 11.62 | 0.7031 | 0.7622 | 0.6524 | 0.6821 |
| 5 | 14.31 | 0.2446 | 0.3041 | 0.2045 | 0.1371 |

The best results among all methods are marked as bold text.

**Table S11. HG002 chr22 small indels detection performance (under the threshold with the best F1 score) for NanoSimFormer simulated signal using different amplitude noise variance**

| Amplitude noise stdev | Variant quality score threshold | F1 | Precision | Recall | Precision-recall AUC |
|---|---|---|---|---|---|
| 0 | 13.64 | **0.8217** | **0.8904** | 0.7628 | **0.7792** |
| 0.5 | 12.63 | 0.8169 | 0.8764 | **0.7649** | 0.7723 |
| 1 | 14.31 | 0.8063 | 0.8880 | 0.7384 | 0.7641 |
| 2 | 10.58 | 0.8046 | 0.8521 | 0.7620 | 0.7675 |
| 3 | 10.14 | 0.7563 | 0.8097 | 0.7095 | 0.7107 |
| 4 | 2.42 | 0.2977 | 0.2281 | 0.4281 | 0.1487 |
| 5 | 6.22 | 0.0565 | 0.0422 | 0.0851 | 0.0075 |

The best results among all methods are marked as bold text.

**Table S12. HG002 chr22 SNPs detection performance (under the threshold with the best F1 score) for NanoSimFormer simulated signal using different event duration variance**

| Event duration stdev | Variant quality score threshold | F1 | Precision | Recall | Precision-recall AUC |
|---|---|---|---|---|---|
| 2.45 | 4.94 | 0.9972 | 0.9972 | 0.9971 | 0.9949 |
| 3.46 | 5.12 | 0.9972 | 0.9970 | 0.9973 | 0.9951 |
| 4.90 | 2.61 | **0.9975** | 0.9971 | **0.9978** | 0.9951 |
| 6.00 | 7.17 | 0.9974 | 0.9974 | 0.9973 | 0.9951 |
| 6.93 | 7.91 | 0.9974 | 0.9974 | 0.9974 | **0.9952** |
| 7.75 | 10.28 | 0.9973 | **0.9975** | 0.9970 | 0.9952 |
| 8.49 | 8.97 | 0.9973 | 0.9975 | 0.9970 | 0.9948 |

The best results among all methods are marked as bold text.

### Table S13. HG002 chr22 small indels detection performance (under the threshold with the best F1 score) for NanoSimFormer simulated signal using different event duration variance

| Event duration stdev | Variant quality score threshold | F1 | Precision | Recall | Precision-recall AUC |
|---|---|---|---|---|---|
| 2.45 | 15.18 | 0.8218 | 0.8800 | **0.7708** | **0.7801** |
| 3.46 | 15.75 | **0.8244** | 0.8983 | 0.7617 | 0.7793 |
| 4.90 | 14.07 | 0.8200 | 0.8842 | 0.7645 | 0.7761 |
| 6.00 | 13.64 | 0.8217 | 0.8904 | 0.7628 | 0.7792 |
| 6.93 | 12.85 | 0.8174 | 0.8866 | 0.7581 | 0.7644 |
| 7.75 | 13.65 | 0.8125 | **0.8989** | 0.7412 | 0.7622 |
| 8.49 | 11.89 | 0.8084 | 0.8757 | 0.7507 | 0.7602 |

The best results among all methods are marked as bold text.

**Table S14. HG002-RNA004 chr22 SNPs detection performance (under the threshold with the best F1 score) for NanoSimFormer simulated signal using different amplitude noise variance**

| Amplitude noise stdev | Variant quality score threshold | F1 | Precision | Recall | Precision-recall AUC |
|---|---|---|---|---|---|
| 0 | 8.66 | **0.9515** | **0.9752** | **0.9290** | **0.9186** |
| 1 | 9.07 | 0.9189 | 0.9329 | 0.9053 | 0.9040 |
| 2 | 10.79 | 0.8732 | 0.8655 | 0.8810 | 0.8775 |
| 2.5 | 12.31 | 0.8012 | 0.8239 | 0.7798 | 0.8174 |
| 3 | 13.21 | 0.6032 | 0.6463 | 0.5655 | 0.5449 |
| 3.5 | 8.39 | 0.0558 | 0.0370 | 0.1136 | 0.0039 |
| 4 | 14.79 | 0.0256 | 0.0455 | 0.0179 | 0.0003 |

The best results among all methods are marked as bold text.

**Table S15. HG002-RNA004 chr22 small indels detection performance (under the threshold with the best F1 score) for NanoSimFormer simulated signal using different amplitude noise variance**

| Amplitude noise stdev | Variant quality score threshold | F1 | Precision | Recall | Precision-recall AUC |
|---|---|---|---|---|---|
| 0 | 13.02 | 0.5789 | **0.7857** | 0.4583 | **0.4712** |
| 1 | 13.32 | **0.6000** | 0.7500 | **0.5000** | 0.4608 |
| 2 | 16.07 | 0.4444 | 0.6667 | 0.3333 | 0.2510 |
| 2.5 | 16.46 | 0.2703 | 0.3846 | 0.2083 | 0.0957 |
| 3 | 17.60 | 0.1875 | 0.3750 | 0.1250 | 0.0458 |
| 3.5 | 14.78 | 0.0588 | 0.1429 | 0.0370 | 0.0002 |
| 4 | 15.70 | 0.0000 | 0.0000 | 0.0000 | 0.0000 |

The best results among all methods are marked as bold text.

**Table S16. HG002-RNA004 chr22 SNPs detection performance (under the threshold with the best F1 score) for NanoSimFormer simulated signal using different event duration variance**

| Event duration stdev | Variant quality score threshold | F1 | Precision | Recall | Precision-recall AUC |
|---|---|---|---|---|---|
| 3.87 | 8.06 | **0.9544** | **0.9874** | 0.9235 | 0.9131 |
| 5.48 | 8.45 | 0.9419 | 0.9809 | 0.9059 | 0.8963 |
| 7.75 | 8.66 | 0.9515 | 0.9752 | **0.9290** | **0.9186** |
| 9.49 | 8.27 | 0.9369 | 0.9630 | 0.9123 | 0.9044 |
| 10.95 | 8.20 | 0.9309 | 0.9627 | 0.9012 | 0.8933 |
| 12.25 | 8.66 | 0.9394 | 0.9688 | 0.9118 | 0.9024 |
| 13.42 | 8.58 | 0.9458 | 0.9813 | 0.9128 | 0.9062 |

The best results among all methods are marked as bold text.

**Table S17. HG002-RNA004 chr22 small indels detection performance (under the threshold with the best F1 score) for NanoSimFormer simulated signal using different event duration variance**

| Event duration stdev | Variant quality score threshold | F1 | Precision | Recall | Precision-recall AUC |
|---|---|---|---|---|---|
| 3.87 | 14.06 | 0.5946 | 0.8462 | 0.4583 | 0.4490 |
| 5.48 | 15.68 | 0.5714 | **0.9091** | 0.4167 | 0.4341 |
| 7.75 | 13.02 | 0.5789 | 0.7857 | 0.4583 | 0.4712 |
| 9.49 | 10.62 | 0.5417 | 0.5200 | **0.5652** | 0.4355 |
| 10.95 | 12.47 | **0.6000** | 0.7500 | 0.5000 | **0.4821** |
| 12.25 | 14.70 | 0.5294 | 0.9000 | 0.3750 | 0.3774 |
| 13.42 | 14.59 | 0.5294 | 0.9000 | 0.3750 | 0.3707 |

The best results among all methods are marked as bold text.

## Table S18. Runtime and memory usage

| Dataset | Simulators | Time (s) | Speed (Mbp/s) | Memory usage (GB) |
|---|---|---|---|---|
| HG002 (chr22) | NanoSimFormer | 3981 | 0.54 | 4.04 |
| | Seq2squiggle | 5893 | 0.37 | 5.89 |
| | Squigulator | 655.81 | 3.26 | 0.32 |
| E.coli | NanoSimFormer | 2075 | 0.54 | 3.00 |
| | Seq2squiggle | 3494.09 | 0.32 | 4.32 |
| | Squigulator | 405.49 | 2.73 | 0.20 |
| KP | NanoSimFormer | 2018.6 | 0.51 | 2.89 |
| | Seq2squiggle | 3354.74 | 0.30 | 3.95 |
| | Squigulator | 224.27 | 4.48 | 0.17 |
| MM | NanoSimFormer | 1584.9 | 0.55 | 2.75 |
| | Seq2squiggle | 2219.24 | 0.39 | 3.85 |
| | Squigulator | 189.36 | 4.47 | 0.16 |
| PA | NanoSimFormer | 1365.48 | 0.53 | 2.58 |
| | Seq2squiggle | 2043.85 | 0.35 | 3.64 |
| | Squigulator | 195.56 | 3.62 | 0.18 |
| PM | NanoSimFormer | 960 | 0.53 | 2.33 |
| | Seq2squiggle | 1803.95 | 0.28 | 3.34 |
| | Squigulator | 108.28 | 4.57 | 0.17 |
| MSA-1010 | NanoSimFormer | 3598 | 0.42 | 4.05 |
| | Seq2squiggle | 4572 | 0.33 | 6.55 |
| | Squigulator | 525 | 2.83 | 2.21 |
| HG002-RNA004 | NanoSimFormer | 1681 | 0.39 | 2.40 |
| | Squigulator | 330.85 | 1.85 | 1.13 |

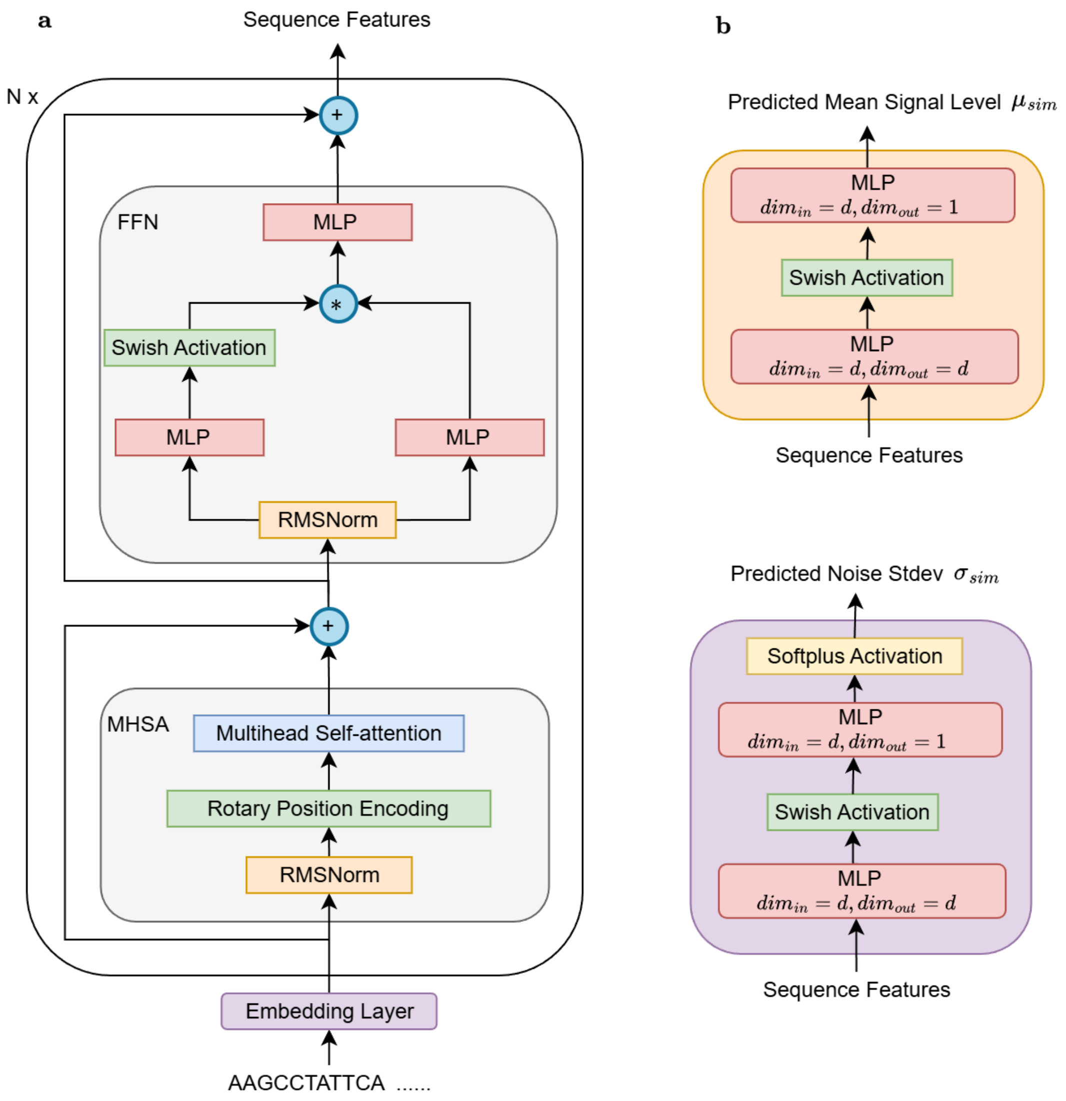


**Fig S1. Detailed architecture of sequence encoder and FFN prediction modules in NanoSimFormer model. a,** Architecture of Transformer-based sequence encoder. **b,** Architecture of Feedforward Neural Network (FFN) for mean signal level and noise standard deviation prediction at the base level.

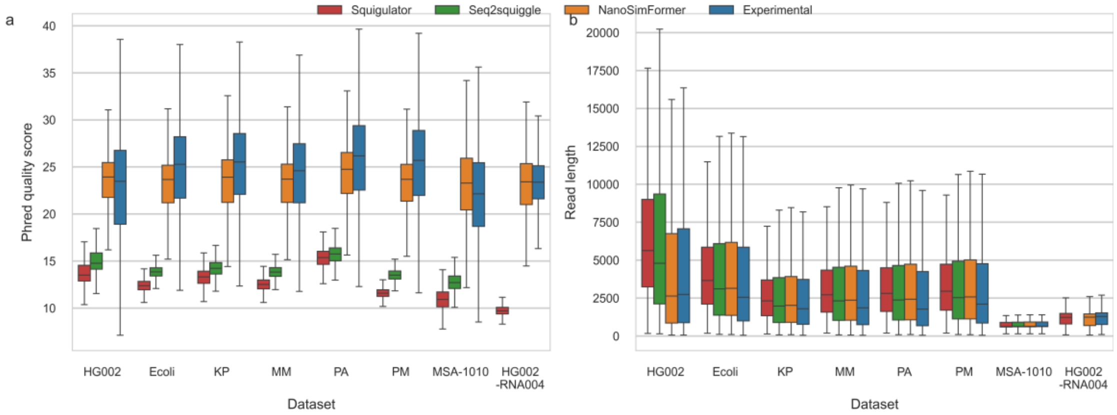


**Fig S2. Basecalling performance of different simulators on various species.** Performance metrics were evaluated for real experimental data (blue) and simulated signals from NanoSimFormer (orange), seq2squiggle (green), and Squigulator (red) across different datasets. The evaluation metrics include: **a,** Phred quality score; and **b,** Read length. In the box plots, the center line represents the median, the edges of the box denote the first and third quartiles, thus enclosing the interquartile range (IQR), and the whiskers extend to the most extreme data points within 1.5x IQR from the edges of the box. Sample reads used to derive these statistics are: HG002 (n=320000), *E. coli* (n=254210), KP (n=365393), MM (n=261589), PA (n=211667), PM (n=140727), MSA-1010 (n=1884844) and HG002-RNA004 (n=528128).

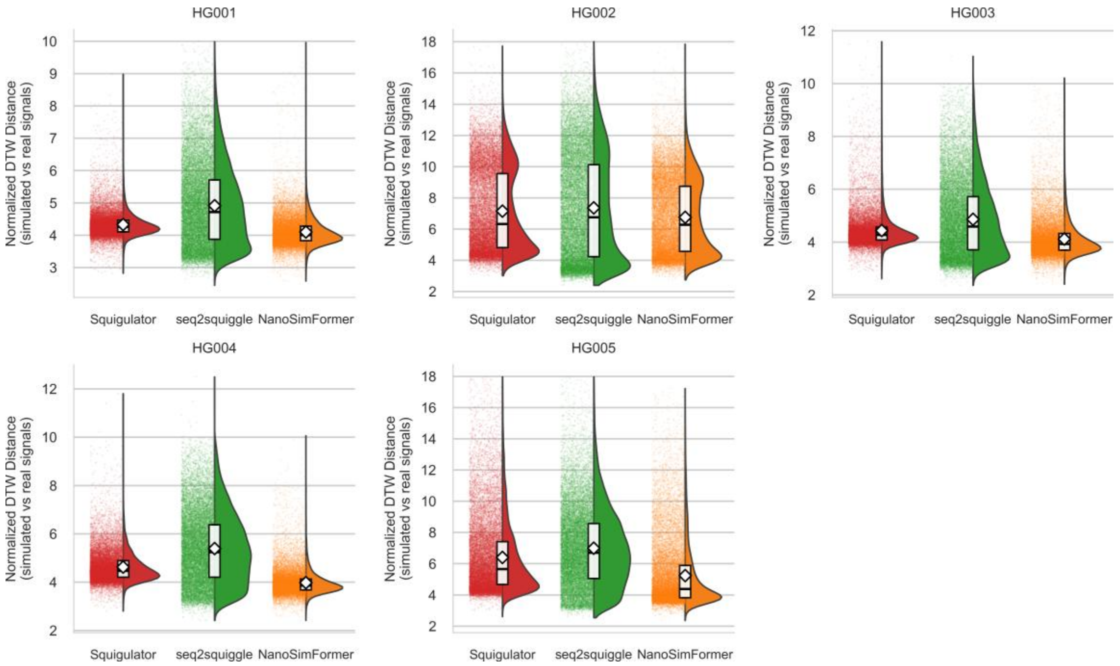


**Fig S3. Comparison of signal-level fidelity between simulated and experimental reads using Normalized Dynamic Time Warping (DTW) distance across five human DNA R10.4.1 datasets.** Raincloud plots combine violin plots that show the distribution of Normalized DTW distances, box plots, means (diamonds), and individual data points. Box plots depict the interquartile ranges and medians (black lines). N=20,000 sampled reads are used to derive statistics for each dataset.

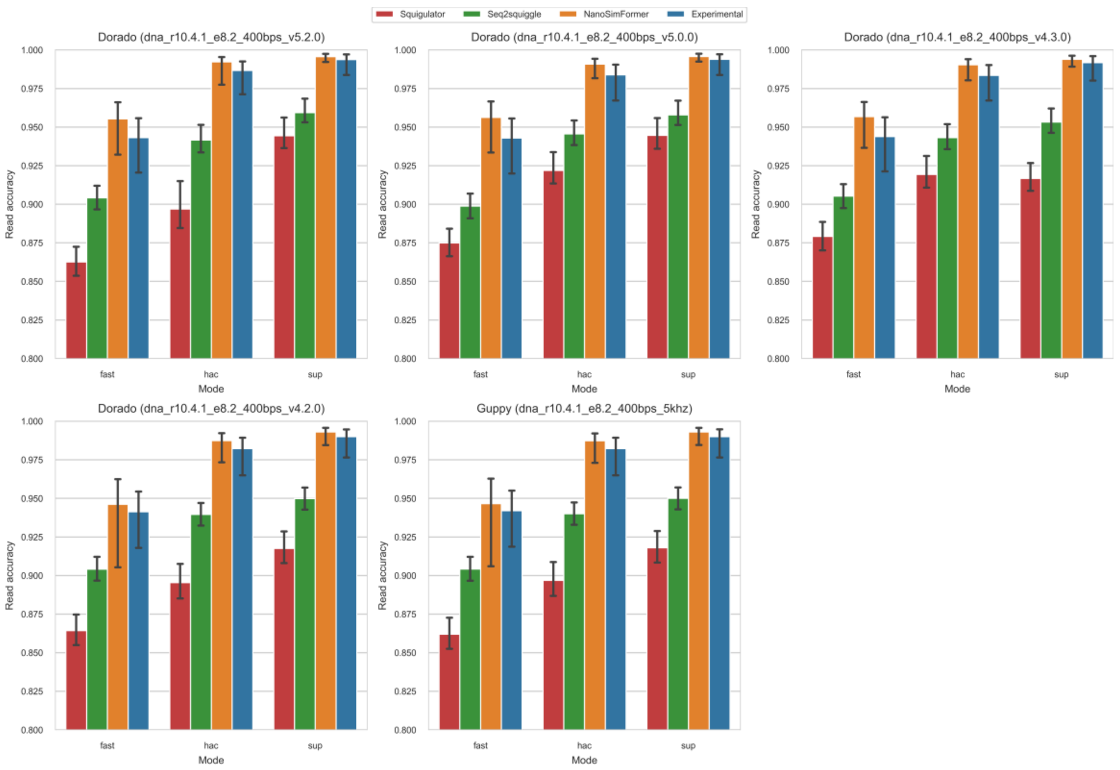


**Fig S4. Basecalling accuracy of experimental and simulated signal across different basecallers and basecalling models on the HG002 chromosome 22 dataset.** Bar charts comparing read accuracy obtained from real experimental reads against simulated reads generated by NanoSimFormer, seq2squiggle, and Squigulator across different R10.4.1 basecalling configurations, including: **Dorado v5.2.0, Dorado v5.0.0**, **Dorado v4.3.0**, **Dorado v4.2.0** and **Guppy**, under three modes (fast, hac, and sup). The height of each bar represents the median read accuracy, and error bars denote the central 50% percentile interval (25th–75th percentiles). Sample reads used to derive these statistics are from the Human HG002 chromosome 22 dataset (n=320,000).

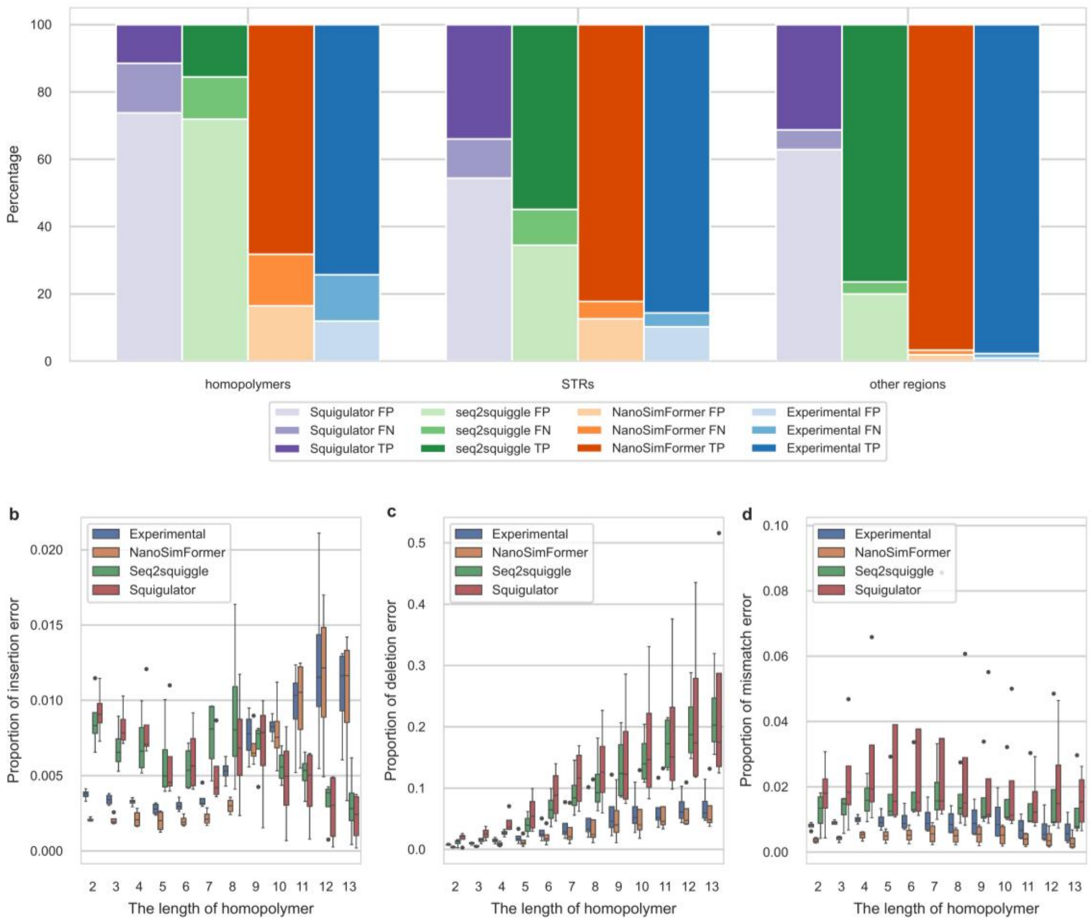


**Fig S5. Percentage of detected small variants stratified by different genomic regions and the corresponding homopolymer error profiles on the HG002 chromosome 22 dataset. a,** Stacked bar chart displaying the percentage of True Positives (TP, darkest shade), False Negatives (FN, medium shade), and False Positives (FP, lightest shade) of detected small variants stratified by genomic context, including homopolymers, short tandem repeats (STRs), and other genomic regions. **b-d,** Insertion, deletion, and mismatch errors for homopolymers of varying lengths on the HG002 chromosome 22 dataset. Error proportions reflect the frequency of insertion, deletion, or mismatch errors occurring within these regions. For all box plots, the center line represents the median, the lower and upper bounds of the box represent the 25th and 75th percentiles, respectively, and the whiskers extend to the minimum and maximum values within 1.5 times the interquartile range, with outliers displayed as individual points. Statistics for all panels were derived from the Human HG002 chromosome 22 dataset with a sample size of n=320000 reads.

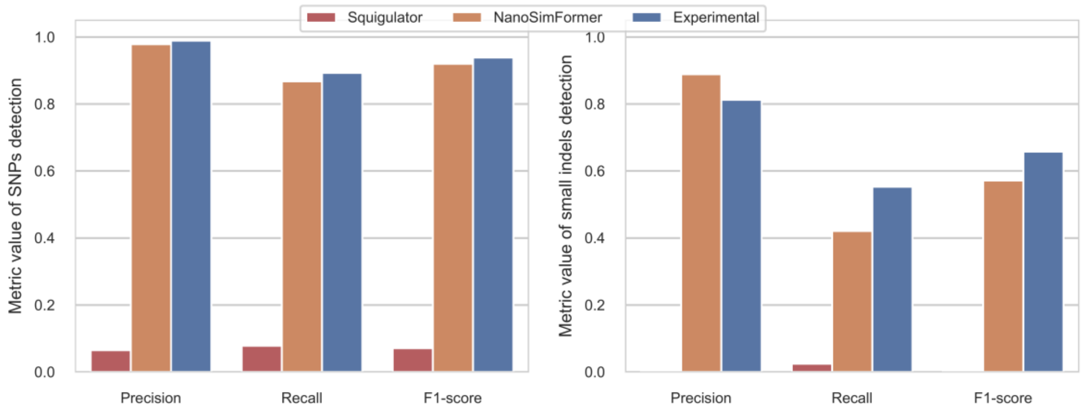


**Fig S6. Bar plot depicting the precision, recall, and F1 score of the small variants (SNPs and small indels) detection performance (under the variant quality threshold with the best F1 score) on the HG002-RNA004 chromosome 22 dataset (n = 528,128 reads).**

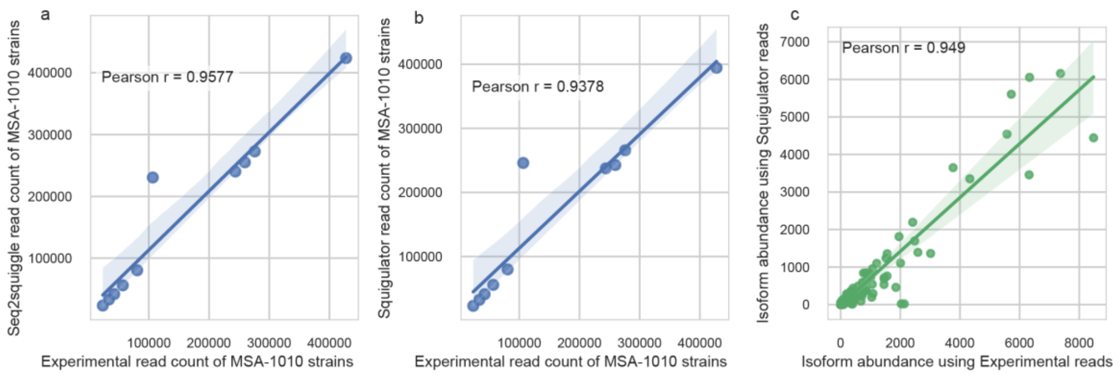


**Fig S7. Abundance preservation in simulated data from seq2squiggle and Squigulator. a, b,** Scatter plots illustrating the correlation of abundance (read counts classified by minimap2) between the real experimental data and the simulated outputs from seq2squiggle and Squigulator for the MSA-1010 fungal mock community, comprising n=10 distinct fungal strains. **c,** Scatter plots showing the correlation between transcript expression (quantified as raw abundance) estimates derived from simulated reads generated by Squigulator versus those obtained from real experimental reads in the HG002-RNA004 dataset. The analysis was performed on n=319 transcripts for Squigulator. For all panels, the solid line represents the linear regression fit, and the shaded band indicates the 95\% confidence interval (CI) of the regression estimate. The Pearson correlation coefficient ($r$) was provided for each comparison.

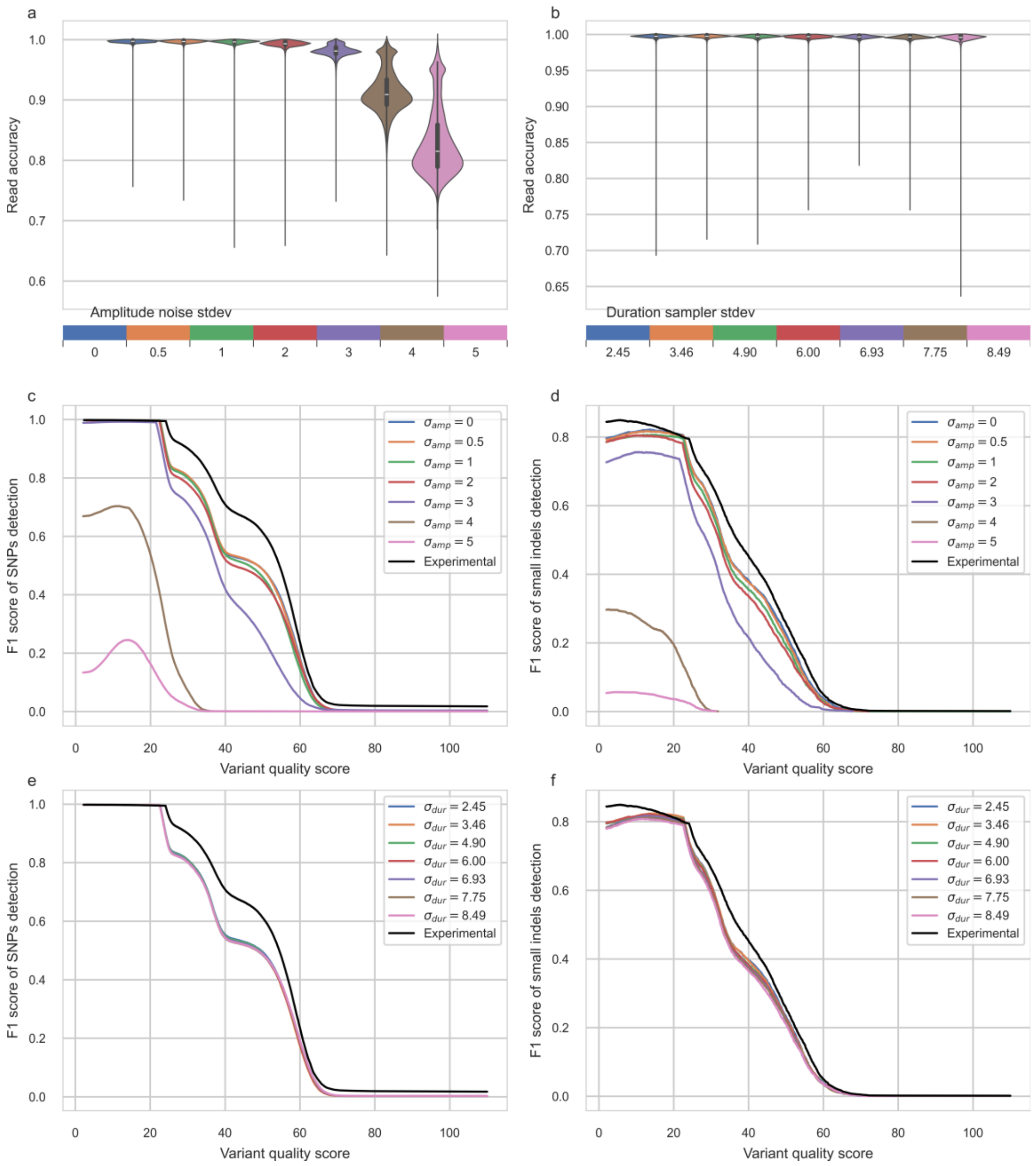


**Fig S8. Impact of amplitude noise and event duration variance on signal fidelity and downstream analysis on the HG002 chromosome 22 dataset. a, b,** Violin plots illustrating the distribution of read accuracy for signals simulated by NanoSimFormer under varying parameters: **a,** amplitude noise standard deviations ($\sigma_{amp}$) ranging from 0 to 5, and **b,** event duration standard deviations ($\sigma_{dur}$) ranging from 2.45 to 8.49. In the violin plots, the internal box plots are defined as follows: the white center point represents the median, the lower and upper bounds of the thick bar represent the 25th and 75th percentiles, respectively, and the whiskers extend to the minimum and maximum values within 1.5 times the interquartile range. **c, d,** F1-score curves plotted against variant quality score thresholds for **c,** SNPs and **d,** small indels under varying amplitude noise intensities. **e, f,** F1-score curves plotted against variant quality score thresholds for **e,** SNPs and **f,** small indels under varying event duration variances. All statistics were derived from the Human HG002 chromosome 22 dataset with a sample size of n=320000 reads.

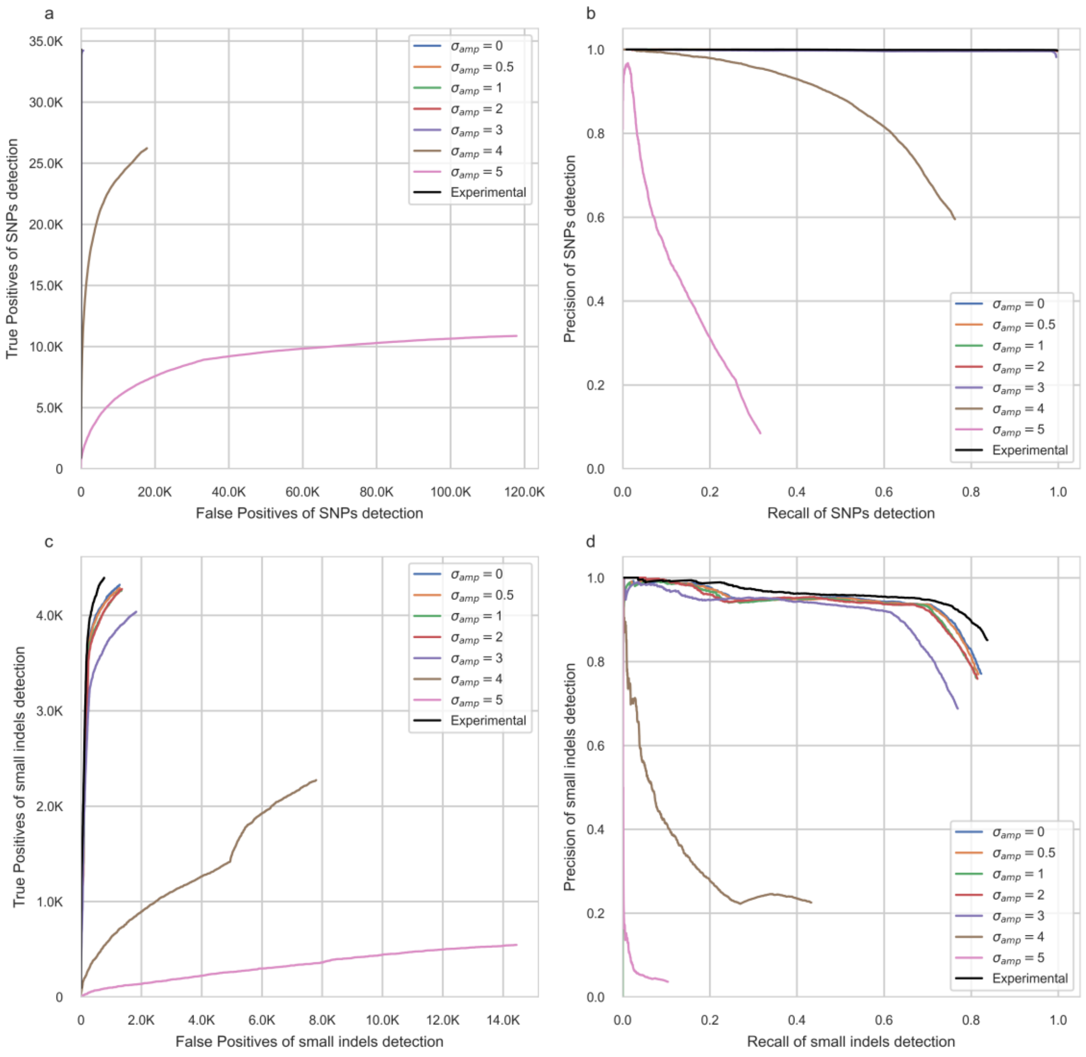


**Fig S9. Small variants (SNPs and small indels) detection performance for NanoSimFormer simulated signal using different amplitude noise variance on the HG002 chromosome 22 dataset. a,** ROC curves for SNPs. **b,** Precision-recall curves for SNPs. **c,** ROC curves for small indels. **d,** Precision-recall curves for small indels.

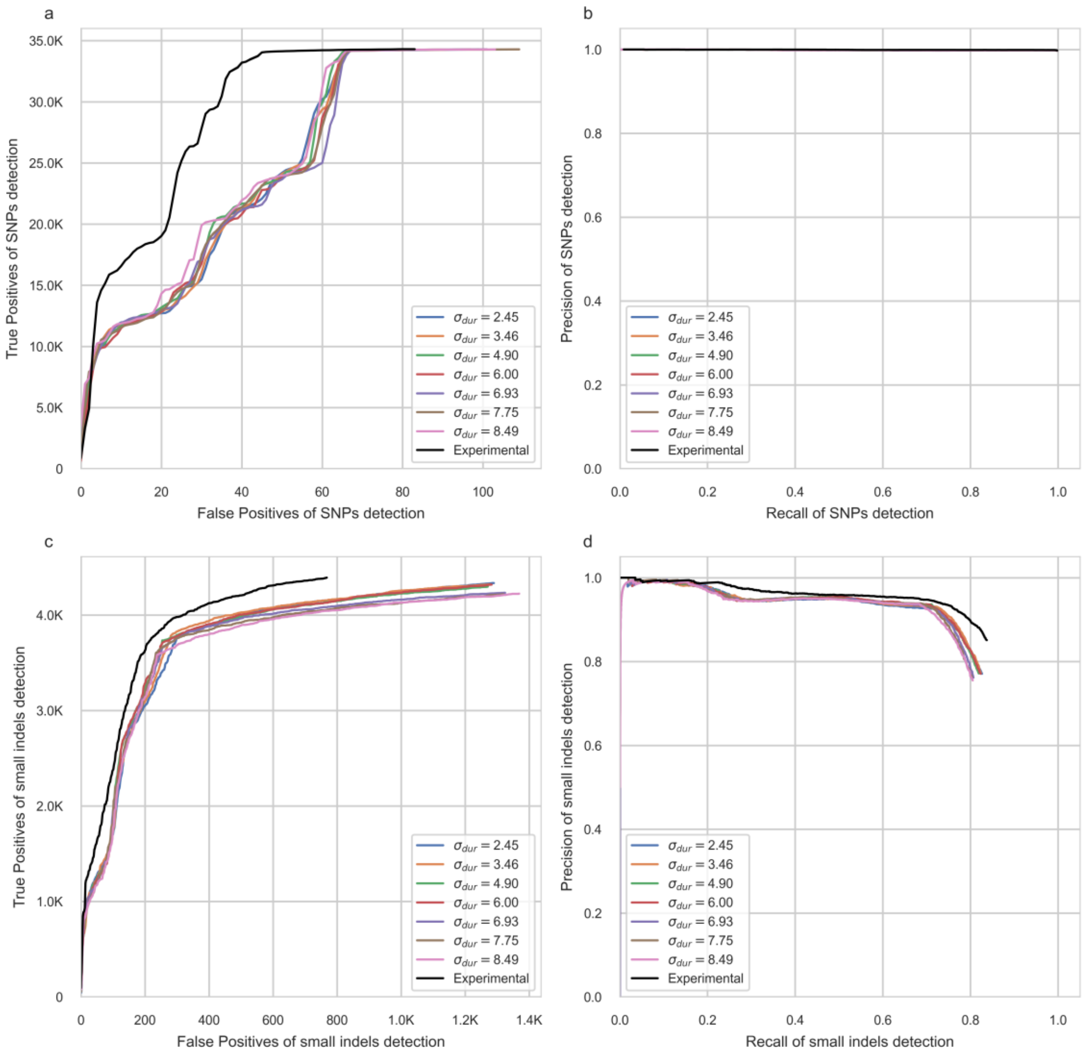


**Fig S10. Small variants (SNPs and small indels) detection performance for NanoSimFormer simulated signal using different event duration variance on the HG002 chromosome 22 dataset. a,** ROC curves for SNPs. **b,** Precision-recall curves for SNPs. **c,** ROC curves for small indels. **d,** Precision-recall curves for small indels.

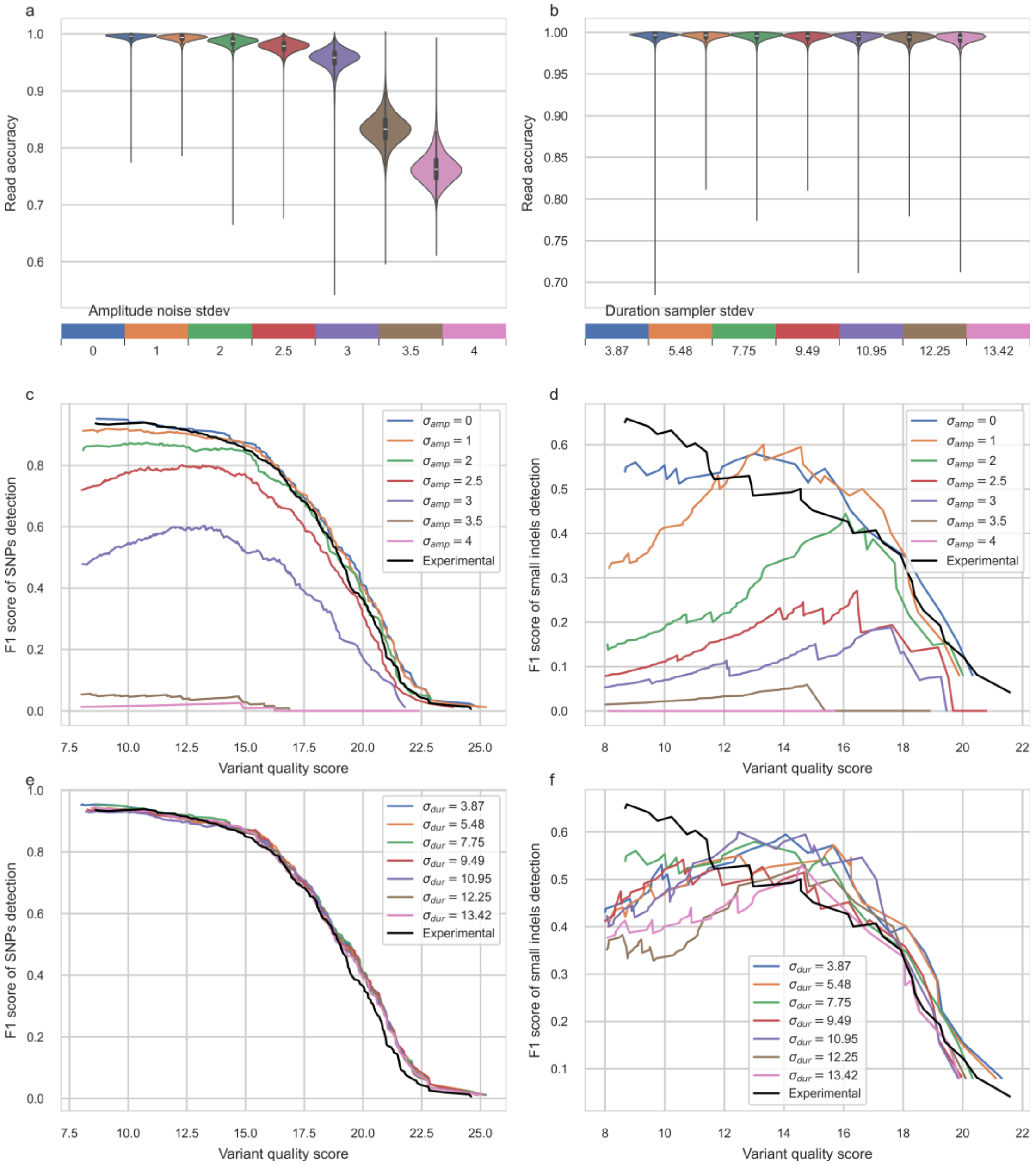


**Fig S11. Impact of amplitude noise and event duration variance on signal fidelity and downstream analysis on the HG002-RNA004 chromosome 22 dataset. a, b,** Violin plots illustrating the distribution of read accuracy for direct-RNA sequencing signals simulated by NanoSimFormer under varying parameters: **(a)** amplitude noise standard deviations ($\sigma_{amp}$) ranging from 0 to 4, and **(b)** event duration standard deviations ($\sigma_{dur}$) ranging from 3.87 to 13.42. In the violin plots, the internal box plots are defined as follows: the white center point represents the median, the lower and upper bounds of the thick bar represent the 25th and 75th percentiles, respectively, and the whiskers extend to the minimum and maximum values within 1.5 times the interquartile range. **c, d,** F1-score curves plotted against variant quality score thresholds for **(c)** SNPs and **(d)** small indels under varying amplitude noise intensities. **e, f,** F1-score curves plotted against variant quality score thresholds for **(e)** SNPs and **(f)** small indels under varying event duration variances. All statistics were derived from the HG002-RNA004 chromosome 22 dataset with a sample size of n = 528,128 reads.

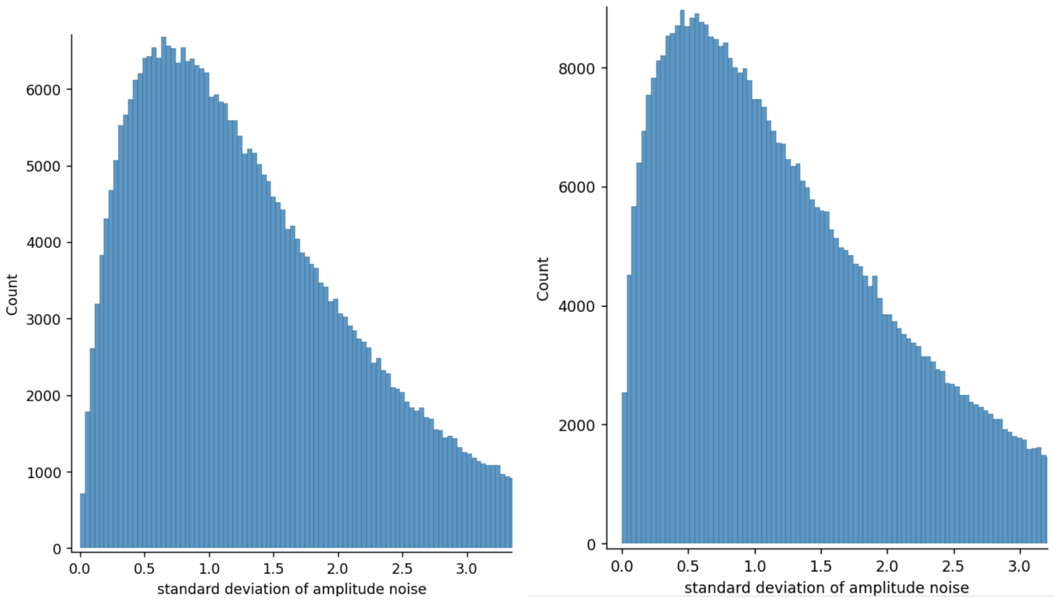


**Fig S12. Gamma distribution of the amplitude noise standard deviation values used in the default simulation setting for R10.4.1 DNA sequencing (left, shape=1.85, scale=0.80) and RNA004 sequencing (right, shape=1.5, scale=1.0).**

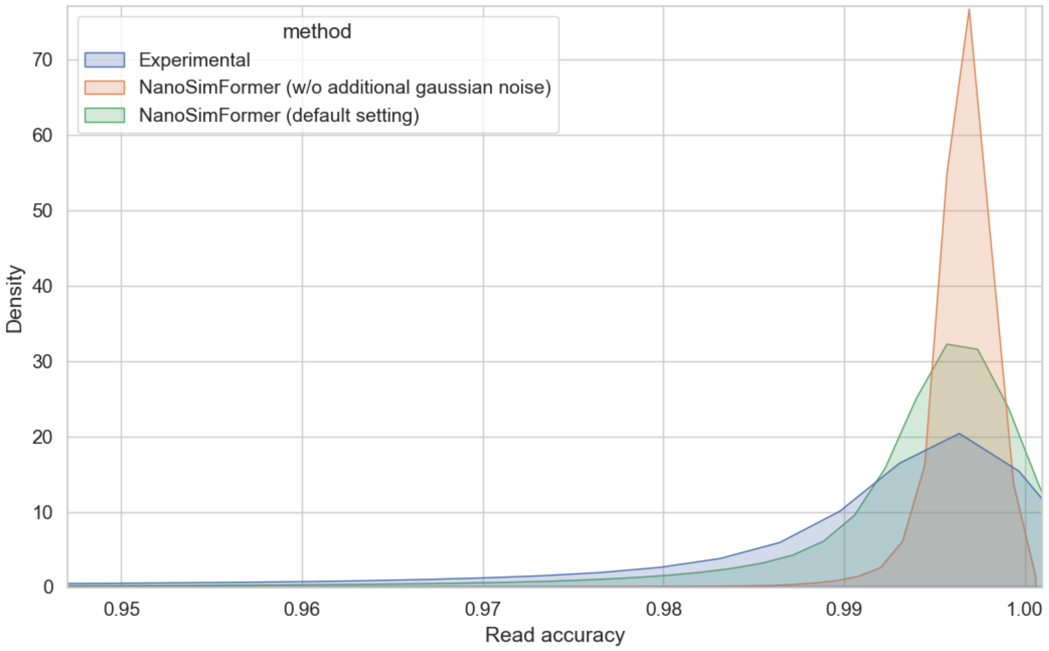


**Fig S13. Effect of default Gamma-sampled standard deviation of per-read amplitude noise on the simulated read accuracy.** Kernel density estimates of read basecalling accuracy for the HG002 R10.4.1 chromosome 22 dataset. Blue: experimental reads. Orange: NanoSimFormer simulation without additional per-read Gaussian noise ($\sigma_{amp} = 0$). Green: NanoSimFormer simulation with the default setting, where per-read noise intensity $\sigma_{amp}$ is sampled from a Gamma distribution.

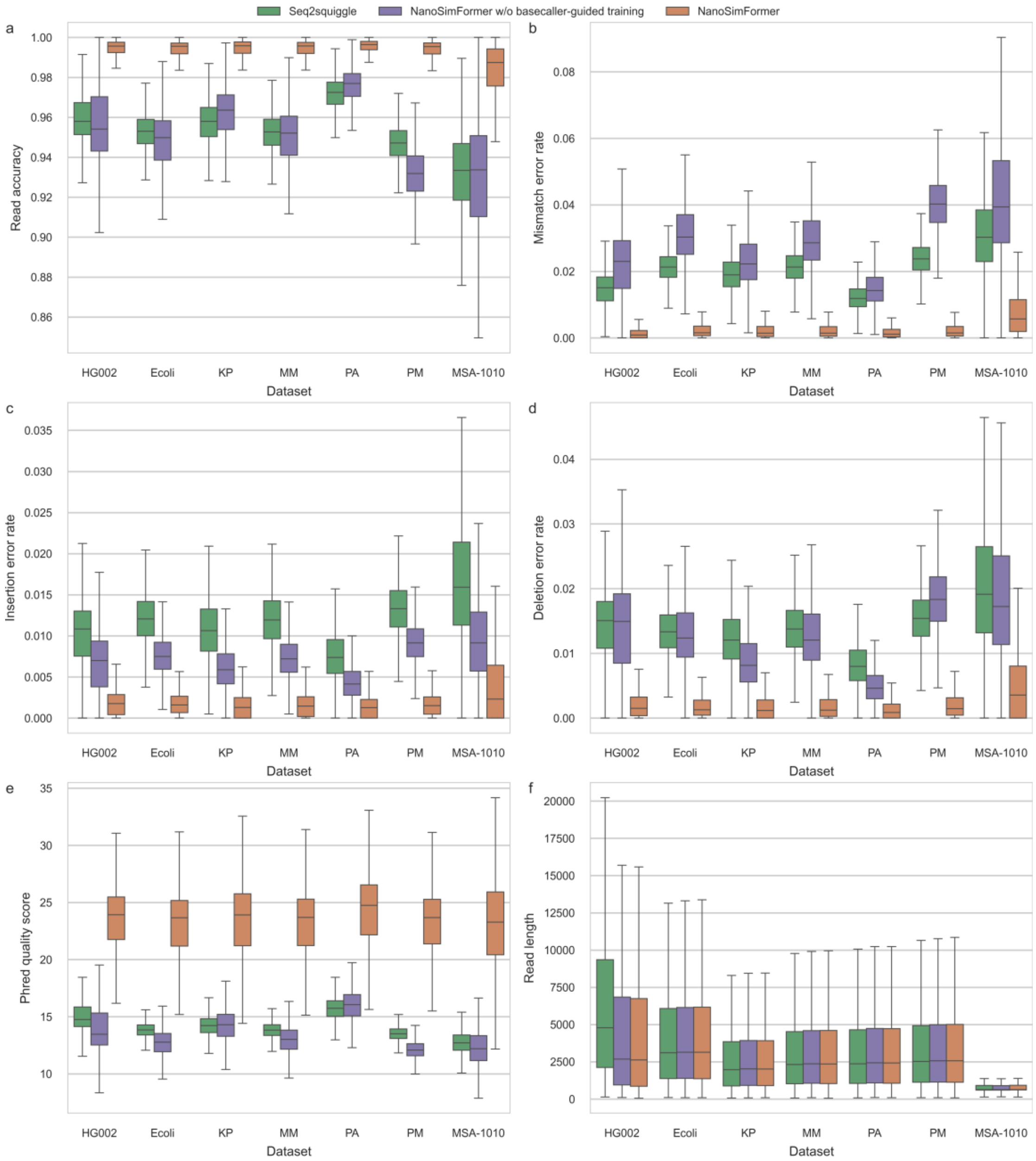


**Fig S14. Basecalling performance of seq2squiggle, NanoSimFormer and its ablated model without basecaller-guided training on various species.** The evaluation metrics include: a, Read accuracy; b, Mismatch error rate; c, Insertion error rate; d, Deletion error rate; e, Phred quality score; and f, Read length. In the box plots, the center line represents the median, the edges of the box denote the first and third quartiles, thus enclosing the interquartile range (IQR), and the whiskers extend to the most extreme data points within 1.5x IQR from the edges of the box. Sample reads used to derive these statistics are: HG002 (n=320000), E. coli (n=254210), KP (n=365393), MM (n=261589), PA (n=211667), PM (n=140727), and MSA-1010 (n=1884844).
